# Unraveling the hierarchical structure of posture and muscle activity changes during mating of *Caenorhabditis elegans*

**DOI:** 10.1093/pnasnexus/pgae032

**Published:** 2024-01-24

**Authors:** Yufeng Wan, Luca Henze Macias, Luis Rene Garcia

**Affiliations:** Department of Biology, Texas A&M University, 3258 TAMU, College Station, TX 77843, USA; Department of Biology, Texas A&M University, 3258 TAMU, College Station, TX 77843, USA; Department of Biology, Texas A&M University, 3258 TAMU, College Station, TX 77843, USA

**Keywords:** calcium imaging, behavior library, behavior structure, muscles, *tph-1*

## Abstract

One goal of neurobiology is to explain how decision-making in neuromuscular circuits produces behaviors. However, two obstacles complicate such efforts: individual behavioral variability and the challenge of simultaneously assessing multiple neuronal activities during behavior. Here, we circumvent these obstacles by analyzing whole animal behavior from a library of *Caenorhabditis elegans* male mating recordings. The copulating males express the GCaMP calcium sensor in the muscles, allowing simultaneous recording of posture and muscle activities. Our library contains wild type and males with selective neuronal desensitization in serotonergic neurons, which include male-specific posterior cord motor/interneurons and sensory ray neurons that modulate mating behavior. Incorporating deep learning–enabled computer vision, we developed a software to automatically quantify posture and muscle activities. By modeling, the posture and muscle activity data are classified into stereotyped modules, with the behaviors represented by serial executions and transitions among the modules. Detailed analysis of the modules reveals previously unidentified subtypes of the male's copulatory spicule prodding behavior. We find that wild-type and serotonergic neurons–suppressed males had different usage preferences for those module subtypes, highlighting the requirement of serotonergic neurons in the coordinated function of some muscles. In the structure of the behavior, bi-module repeats coincide with most of the previously described copulation steps, suggesting a recursive “repeat until success/give up” program is used for each step during mating. On the other hand, the transition orders of the bi-module repeats reveal the sub-behavioral hierarchy males employ to locate and inseminate hermaphrodites.

**Significance Statement**

Studying complex behavior such as *Caenorhabditis elegans* mating behavior starts with dissecting the behavior into multiple steps. Such classification is conducted by the researchers observing instances of the behavior and therefore may not capture subtle but underlying motor patterns. Here, we combined calcium imaging, computer vision, and Bayesian modeling, enabling the computer to automatically identify and dissect copulation behavior into units that we call modules. Posture and muscle activity data were used and preserved throughout this analysis. Through this method, the variation of a mating step was demonstrated by the combination of different modules. We also discovered the behavior is organized into various bi-module repeats as the basic structure of the behavior.

## Introduction

**Fig. 1.**
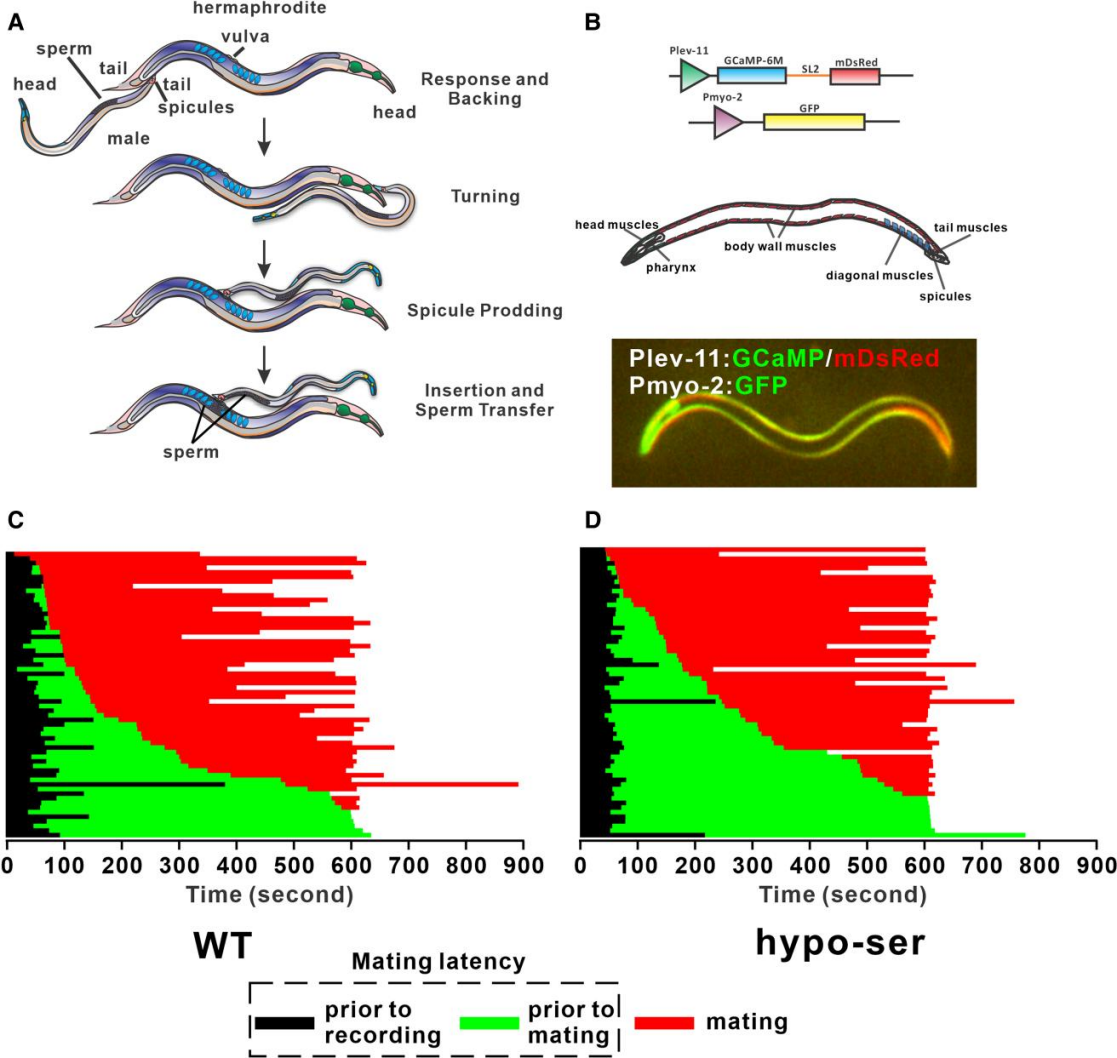
Generating a mating behavior library database. A) Schematic of canonical steps of mating. B) Transgenic worms allowing the monitor of both posture and muscle activity. Top two panels: schematic of plasmids used to generate the transgenic worms. Bottom two panels: schematic of the muscles males use during mating, and an example micrograph showing a fluorescing male with green and red fluorescence overlaid. Mating latency of wild-type (C) and hypo-ser (D) males in the video library. Each horizontal line represents a male in the video library. The length of the color-coded segments represents the time that an animal spent in each corresponding behavior. All data of the same genotype are stacked and sorted based on total time prior to mating to help visualize the distribution.

The male mating behavior of *Caenorhabditis elegans* is a stereotyped behavior and has been described in extensive detail (1–3). The males are most potent to mate as young adults immediately after their last larva molt (4). Under light microscopy observation, the behavior can be dissected into multiple easily distinguishable steps, corresponding to the stepwise strategy to achieve the male's goal of inseminating the hermaphrodite (Fig. 1A): (i) the male responds to the hermaphrodite by pressing its tail ventrally against the partner's body and moving backward, scanning for the vulva. (ii) If the male reaches the end of the hermaphrodite, its tail bends resulting in a sharp ventral arch and a turning of the orientation of the tail, followed by scanning the vulva on the other side of the hermaphrodite. (iii) Once the male finds the vulva, it stops the backing and starts rhythmically thrusting its spicules located at the tail into the vulva. (iv) After the spicules partially penetrate the vulva, the male fully extrudes the spicules inside the vulva, anchoring itself on the hermaphrodite, followed by the transfer of sperm into the hermaphrodite.

Despite the complexity of the behavior, the pioneer work of Hodgkin (5) systematically characterized the effects of gene mutations on the behavior-associated anatomical features and the mating behavior itself, using the number of cross progeny sired by males as the readout for mating efficiency. Later, essential sensory and motor structures were identified through cell ablation combining ethological observations (1, 6, 7). Around the same time, genes essential for the behavior were identified by mutant screenings for easily identifiable defects in mating steps such as response and vulva location (8–11), backing and turning (7, 10, 12), or abnormal behaviors such as spontaneous spicule protraction (6, 13–15). In addition to the essential genes, these studies were able to demonstrate the circuit pathways perturbed by the gene mutations/cell ablations, thanks to the availability of the full connectome (16–19).

In contrast to those seemingly rigid mechanisms and pathways, prevalent variance among mating behaviors of individuals has been noted since the beginning (5). Such variance is demonstrated by the wide range of certain mating statistics among wild-type males, including time spent before (64–264.8 s) or during (4.6–24.2 min) mating (20), number of vulva contacts (1–7 times) before insertion (21), and the frequency (5.9–8.5 Hz) of spicule prodding (6). The variability of the behavior despite the controlled lab condition suggests the mating behavior is highly responsive to various stochastic external and internal factors. Indeed, in the assays assessing the excitability of the copulation circuit, various biological/abiological perturbations of different systems were shown to affect the readout of the experiments (22). Furthermore, the experience of mating itself (or the lack of) can have an impact on subsequent matings (23). These findings suggest that variability is a feature that likely underlies the regulatory mechanisms to coordinate the reproductive behavior with other necessary behaviors.

The structural basis of behavioral variability arises from the extensive connectivity of the connectome: various neural circuits are interconnected to process the information from different sources, constantly adjusting the mating behavior with the fluctuation of internal and external signals (24). Recent developments in whole-brain calcium imaging and automatic neuron identification showed promising results in correlating specific worm behavior to neuronal activities (25–30). However, the identification and tracking of over 300 tiny neurons at high magnification of a moving animal present a high requirement for imaging/tracking and postrecording analysis. Because of this, the integrity of data and throughput of the analysis are limited to generate enough data to understand the deterministic and variable parameters of the animals' behavior (26).

In contrast to the relatively small individual size and hard-to-identify neurons, the muscle system of *C. elegans* male is relatively easy to distinguish (31–33). For the mating behavior, the relevant muscles include the following: those for steering and propelling the body during locomotion (head muscles and body wall muscles); those for bending the tail ventrally (posterior one-third of the body), to press it against the hermaphrodite during mating (diagonal muscles); those for controlling the in-and-out movements of copulatory spicules (spicule muscles); those for controlling the movement of the very end of the tail (oblique muscles); and other accessory muscles in the tail (gubernaculum muscles, sphincter, and anal depressor; Fig. 1B). Theoretically, a segment of behavior can be fully reconstructed by quantifying the starting status and the subsequent changes of those muscles. Also, with the connectome information, one can possibly even propose and test the neuronal activity scenarios that give rise to the patterns of muscle activities, which may underly the decision-making mechanisms of the males during mating.

To fully quantify the mating behavior, a whole worm digital recording is required for understanding the sequence of events that define each individual's behavior. Among the various worm behaviors, the locomotion behavior is extremely simple. As such, multiple “trackers” have been developed to automatically track, identify, and extract posture features of free-moving worms (34). However, currently, no system has been described to deal with the complex posture changes caused by the interaction between the two sexes during mating or track the whole animal's neuromuscular activity with high enough resolution.

Here, we describe an analysis pipeline using a *C. elegans* male mating behavior library to uncover the hierarchy of motor patterns used in a complex behavior. We first generated transgenic worms that allow us to record muscle activities and posture changes during mating simultaneously. Second, we designed a software package called neural network-based automatic worm analyzer (NAWA) to quantify those muscle activities and postures accurately and automatically in the videos. Last, these biological data were modeled using a Bayesian inference method. We applied the same processes with wild-type and serotonergic neurons–suppressed (hypo-ser) males. The model identified various population-shared modules. Each module was defined by an ordered change of posture and muscle activities, representing a programmed activation of several neuromuscular circuits. We unexpectedly found that as many as five different modules are involved in the prodding step of the mating behavior, two of which are differentially used by wild-type and hypo-ser males during mating. Interestingly, the modules are also organized into bi-module repeats for the majority of the behavior. Twelve bi-module repeats form most of the routines that males use to execute the different steps of mating. By comparing the wild-type’s and hypo-ser's usage timing and frequencies of the routines, we were able to partially describe the behavioral differences of the two genetically different populations. We were also able to identify and analyze the relevant behavioral modules and their associated routines that were affected by serotonergic neurons' activity. These behavioral structures can be future investigation focuses to understand the serotonergic neurons function in shaping the decision-making during mating.

## Results

### Generating a digital library of male mating behavior

To monitor the change of muscle activity during mating behavior, we generated a transgenic strain expressing the calcium sensor GCaMP6 with a transcriptionally coupled, SL2 trans-spliced monomeric DsRed (mDsRed) in the muscles by the tropomyosin promoter P*lev-*11 (22, 35). The mDsRed signals can be used to calibrate the expression level of GCaMP6 as well as to outline the shape of the worm independent of muscle activity. The transgene also contains a marker gene expressing green fluorescent protein (GFP) in the pharynx from the myosin promoter P*myo-2* (Fig. 1B). This strain allows us to monitor the muscles' activities as well as the posture changes of the males during mating using a fluorescent microscope. To facilitate the recording and reduce the variance, we made mating arenas by putting 10 paralyzed *unc-64(lf)* hermaphrodites on a ∼5-mm diameter OP50 lawn. These hermaphrodites formed a circular wall so that when a male is put at the center, no matter which direction it moves, it will have an equal chance of encountering a hermaphrodite (Fig. S1A). After a male was placed in the mating arena and located under the microscope, we recorded the male for up to 10 min until it finished or quit mating, representing the first mating event. Considering the variance among individual males, our library contains 62 recordings of day 1 adult wild-type virgin adult males. In this library, both the GCaMP and the mDsRed signals of the males were captured using a fluorescent microscope equipped with a camera mounted on a Dual View Simultaneous Imaging System (Fig. S1B).

As a comparison with wild-type males, we generated transgenic males (hypo-ser) expressing *unc-103(gf)* in serotonergic neurons by the *tph-1* promoter (5, 36, 37). Expression of this mutant K^+^ channel *unc-103(gf)* would hyperpolarize the serotonergic neurons and reduce their sensitivity to excitation. We similarly made a library of 63 recordings with day 1 adult hypo-ser males. From the recordings, actively contracting muscles and their consequences on the posture can be distinguished by the increasing intensity of the GCaMP signal relative to the mDsRed signal and the overall body posture changes outlined by the fluorescing body walls, respectively. Therefore, we generated a combined library containing body posture and corresponding muscle activity data of wild-type and hypo-ser males.

There is a delay between when a male is put into the arena and when it locates a hermaphrodite and starts mating. This mating latency is indicative of a male's copulation drive (20). Despite our attempt in limiting variables, we still observed great variability among wild-type individuals in mating latency from essentially zero to the full length of the experiment, 10 min (Fig. 1C). For the hypo-ser males, the average mating latency is longer than wild type, but the level of variability among individuals is similar to that of wild type recordings (Fig. 1D). The variable mating latency changes the proportion of mating frames vs. nonmating frames in each recording and may impact the behavior during mating. These results confirm the necessity of a large library to capture the complete spectrum of mating behaviors within the same genetic background.

### Automatic posture and muscle activity profiling

Because of the male's constant changing postures during recordings, quantification of muscle activity and posture requires frame-by-frame tracing of the male's outline. Currently, no publicly available software is suitable or capable of accurate automatic tracing of the fluorescing mating *C. elegans* male's profile. Part of the reason is that unlike simple locomotion behavior, decoding the complex body postures exhibited during mating requires a sophisticated algorithm. To accomplish this, we developed a software package, NAWA, to extract data on posture and muscle activities (Fig. S1C and Supplementary Methods). The core of NAWA is a series of trainable deep neural networks (DNNs), which learn to pixel-wisely label the different parts of the worms from the fluorescent images. To train the DNNs, we randomly picked 100 frames from each of the videos and manually labeled the different worm parts. Subsequent parts of NAWA then model the posture of the worm based on that information. With a tiny fraction of the wild-type dataset as the training data (1.72%), the NAWA achieved on average >95% success rate in modeling the entire wild-type dataset as well as >85% success rate in the hypo-ser dataset (Fig. S2A). In both datasets, the majority of the failed frames have insufficient data, either because the manual tracking did not capture the whole male or the male crawled into some imperfections on the arena that distorted the light. Erroneous modeling only accounted for about 5 and 7% of the total frames in the wild-type and hypo-ser datasets, respectively (Fig. S2B). Those frames contain blurred images or overlapping body parts, which are difficult if not impossible to quantify manually (Fig. S2C). These results demonstrate that NAWA's performance is on par with a human's, and the system is capable of analyzing males with different genetic backgrounds without the need to retrain it. For the following analysis, we discarded the defective frames or mathematically interpolated data using neighboring frames (see Supplementary Methods).

**Fig. 2.**
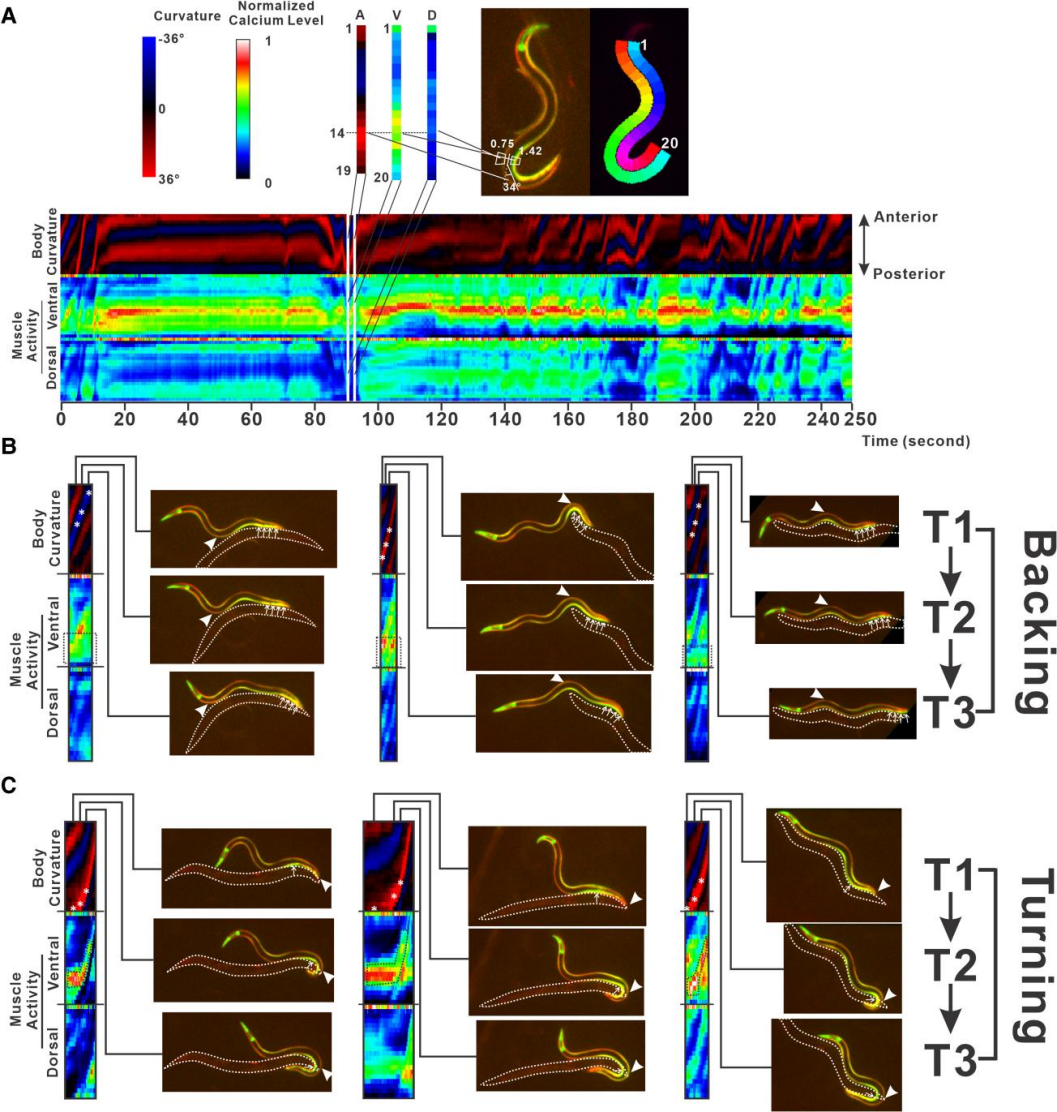
Visualization and validation of data integrity in the database. A) Color-coded visual representation of posture and muscle activity data. Bottom: a 250-s sample block of color-coded posture and muscle activity data. Top left: color codes for curvatures and calcium levels. Top right: a single frame example to illustrate the data arrangement and values stored in the visualization. Nineteen posture angles (A), 20 each ventral muscles activities (V), and dorsal muscles activities (D) data are shown as three color columns on the left. Overlaid fluorescent micrograph and NAWA-generated segments quantification areas are shown on the right. Different ventral and dorsal segments are differentially color labeled for clarity. B) Three samples of backing events. Color-coded data are on the left. Three snapshots of the fluorescent males during these events are on the right. Asterisks demonstrate the anterior transition of the most bent segment, corresponding to the arrowheads in the micrographs. Dashed boxes demonstrate the constant active ventral posterior segments, corresponding to the arrows in the micrographs. C) Three samples of turning events. Color-coded data are on the left. Three snapshots of the fluorescent males during these events are on the right. Asterisks demonstrate the extreme ventral curvatures shifting from the posterior end to anterior segments, corresponding to the arrowheads in the micrographs. Dashed regions demonstrate the shifting of extremely active ventral posterior segments, corresponding to the arrows in the micrographs.

Along the anterior–posterior (AP) axis, 95 body wall muscles are arranged in 4 longitudinal bands called quadrants, with 3 containing 24 muscles and 1 containing 23 muscles (31, 33). Since the GFP fluorescence in the pharynx would greatly affect the quantification of GCaMP in the region, we started anterior quantification at the end of the pharynx, which leaves about 20 body wall muscle segments for each quadrant. Therefore, we segmented the male into 20 equal segments along the AP axis, with each segment approximating 1 set of the 4 muscles in the quadrants. The posture is thus represented by the estimated curvatures between the numbered segments (20 segments with 19 curvature measurements). Ventral and dorsal muscles activities of each segment can be quantified by the ratio between GCaMP and mDsRed fluorescence of the segmented regions. Plotting these quantifications against time illustrates the fluctuations of muscle activities and body curvatures (Fig. 2A). We extracted sample bouts of backing and turning from the videos and matched them with the corresponding quantification data. Through comparison of the samples, apparent behavioral signatures were captured by the quantifications: during backing, the male glides backward with the forward propagation of body curvatures and sustained the activity of diagonal muscle located at the posterior-ventral part of the body (segments 15–20; Fig. 2B). During turning, the male slows down when its tail approaches the end of the hermaphrodite and then ventrally curves its tail to reach the other side and continues to glide backward while forward propagating the ventral curvature. Compared with backing, the most active ventral muscle groups are more anterior toward the middle of the worm (segments 13–18; Fig. 2C). We speculate the difference is because during turning, less contact surface between the male tail and the hermaphrodite requires finer control of the tail tip contraction, avoiding overcurve and losing the contact with the hermaphrodite. These results demonstrate NAWA as one of the more powerful software tools in automatic tracking of the dynamic *C. elegans* postures and muscle activities as shown in the male mating behavior.

### Translating the male mating behavior using a hierarchical structured model

**Fig. 3.**
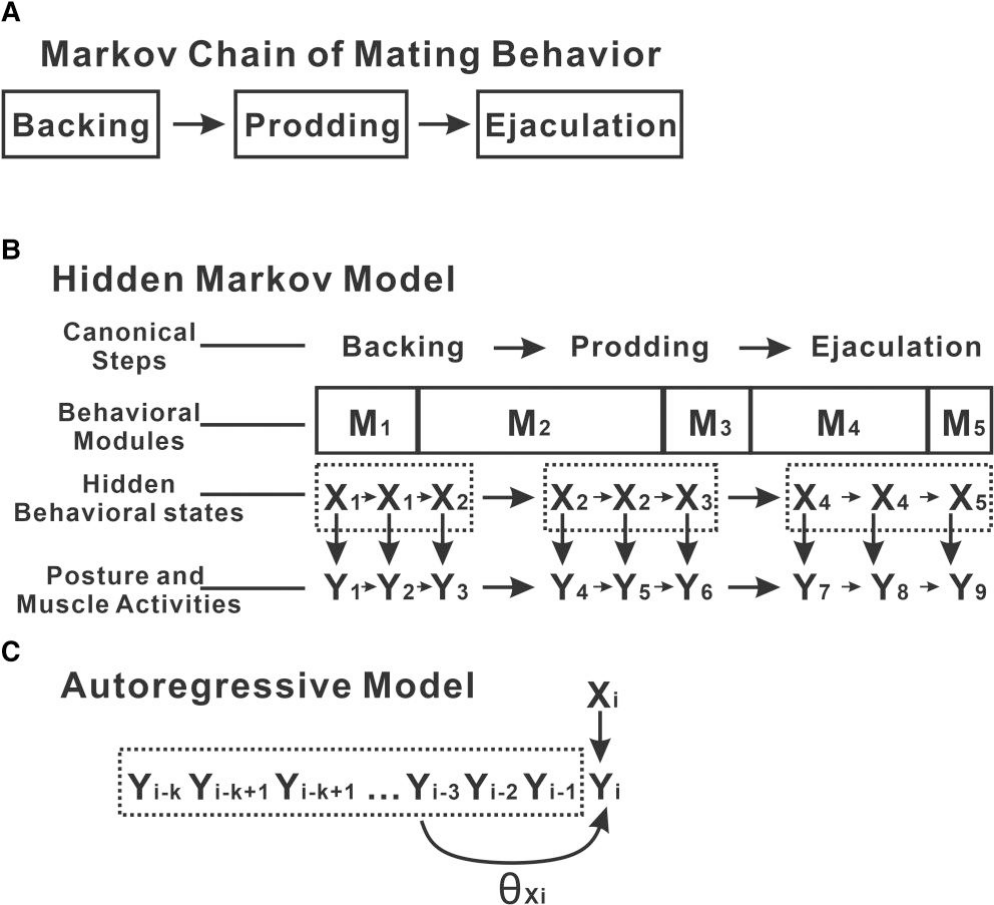
Models explaining the hierarchical structure of mating behavior. A) A Markov chain using the canonical mating steps. B) Illustration of the same behavior in (A) explained using a HMM. In this model, the behavior is explained using a string of behavioral states that follow a Markov model. The behavioral states are not directly observable. Instead, they are inferred from observed posture and muscle activities. The strings of the same behavioral states are grouped into module blocks, which are alternative units of the behavior to the canonical mating steps. C) Illustration of the autoregressive model that defines and distinguishes different modules.

Researchers dissected *C. elegans* male mating behavior into multiple stereotyped steps. The sequence of the steps can be viewed as a Markov chain: during a transition, the previous step determines what could be the next step (Figs. 1A and 3A). However, explaining the mating behavior with this model lacks detailed sophistication and is highly arbitrary, because it requires subjectively assigning the behavior into a very limited set of steps and disregarding the variance within a specific step. Here, we segmented the behavior into nondescriptive modules using a nonparametric method. The modules were identified by structuring the mating behavior using an autoregressive-hidden Markov model (AR-HMM). This is a hierarchical classification of behavior. On a larger scale, the male is transitioning from one state to another or to itself following a Markov model. The blocks of the self-transitioning states form behavioral modules, as alternative units of mating behavior to the arbitrary mating steps. On a smaller scale, each behavioral state activates specific neuromuscular circuits, thus determining the pattern of body postures and muscle activity changes. This pattern can be described in an autoregressive model (Fig. 3B and C). Even though the chain of states is not observable (thus hidden), the body postures and muscle activities are observed and used to infer the chain of states during model fitting. Therefore, the behavioral states are inferred using the observation data during the fitting process, avoiding arbitrary defining of the behavioral events (see Supplementary Methods).

Before fitting the model, we need to perform some model-free analyses and data transformation to set up the model, reflecting the temporal structure of the behavior and increasing the efficiency of the fitting process. Our quantification of posture and muscle activities generated 59 measurements for each frame of recordings (Fig. 2A). We performed principal component analysis to reduce the dimensionality of the data. The first 30 principal components (PCs) explain >90% of the variance in the data and thus were chosen for further modeling (Fig. S3A). Modeling using the PCs instead of the raw data reduced the model size by ∼75%. We also performed three model-free analyses to gain insight into the temporal structure of the data. First, we performed autocorrelation analysis, which quantifies the average correlation of data points to the preceding data points at different lags (38). This analysis tells us how temporally sustained the data are and how long it takes for the data to be unpredictable due to behavioral changes. In both wild-type and hypo-ser datasets, the autocorrelation decreased sharply in about 2 s, indicating the data progressively deviated from the beginning in this time frame. However, the autocorrelation remained high at 0.4–0.5, suggesting there is a boundary to the deviation. After that, the decline became gradual and remained at 0.3–0.4 after 10 s, suggesting that a different mode of deviation from the first 2 s is changing the behavior (Fig. S3B). The two tiers of autocorrelation decrease may correlate with the layers of behavioral structures mentioned before. The smaller scale behaviors were explainable by autoregression models, capturing the fast progressive deviation of data. The larger scale behaviors were explainable by HMM, modeling the infrequent change of behavioral modules. Second, we analyzed the behavioral frequency signatures in the data using Welch's power spectral density estimate. This analysis identifies the periodic components in the data using the Fourier transform (39). The periods of those components inform us of the time between males repeating their behavior, which corresponds to the length of the behavioral unit. Most of the signal components for both wild-type and hypo-ser datasets concentrated between 0.12 and 1.20 Hz (Fig. S3C), suggesting that the length of most behavioral units extends between 0.8 and 8 s. Last, we estimated the sizes of behavioral blocks by approximating boundaries between blocks with a simple algorithm called the changepoint algorithm, which detects when data suddenly change (40). While we do not believe this algorithm can reliably identify the behavioral units, we reasoned that sudden changes detected by the changepoint likely coincide with decisional changes in the behavior. Therefore, statistical distributions of the time between sudden changes in data should approximate the temporal dynamic of the behavior. The ranges of the behavioral block sizes estimated using such methods were between 0.3 and 32 s, with the majority of the blocks concentrated between 0.4 and 5 s (Fig. S3D and E). This range is in line with the range estimates from the previous two analyses. As a result, we used these data as a reference in tuning the module sizes during the model fitting process.

The AR-HMM model was fit through a Bayesian approach, which infers the module identification for each frame in the data. In this process, the autoregressive parameters that define each module and the transition probabilities among modules were determined. However, the duration of each behavioral module is sensitive to the self-transition bias parameter kappa. Without this bias parameter, the model would generate unrealistic rapid module transitions (41, 42). Gradual increase of kappa reduces module transition probabilities and results in average longer durations of behavioral modules. By testing different kappa values and comparing the results, we optimized the model, so that the module size distributions match the behavioral block sizes generated by the changepoint algorithm (Fig. S3D–D’’’ and E–E’’’ and Supplementary Methods).

### Modeling reveals novel behavioral modules associated with canonical mating steps

**Fig. 4.**
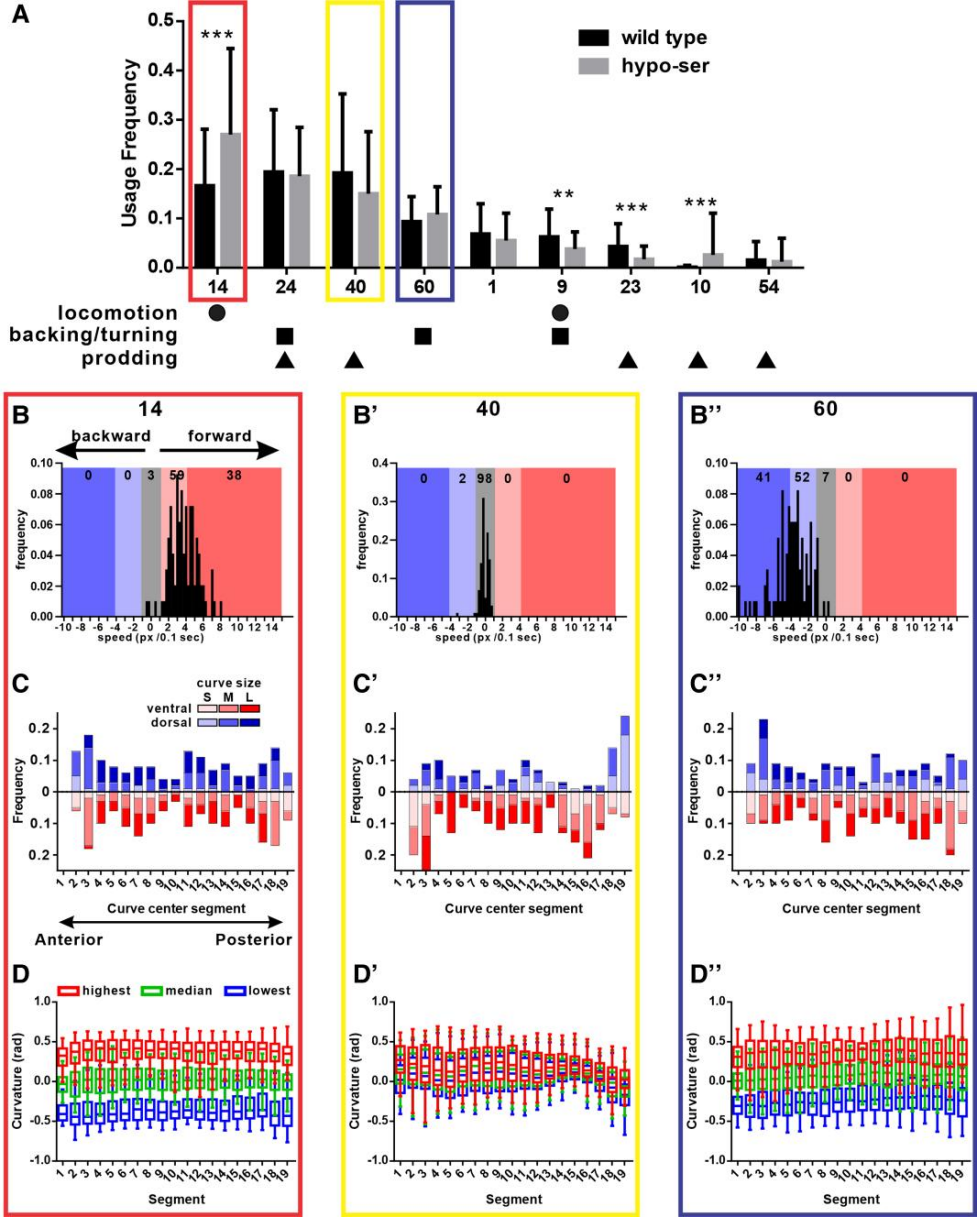
Characteristics of major behavioral modules. A) Usage frequencies of major behavioral modules among all the recordings by wild-type and hypo-ser males. The different shapes label the mating steps that are associated with those modules. The error bars represent standard deviation. *P*-value is calculated using the Mann–Whitney *U* test. **P < 0.01 and ***P < 0.001. *n* equals 62 and 63 for wild-type and hypo-ser males, respectively. Speed and posture characteristics of a locomotion module 14 (B, C, and D), a prodding module 40 (B’, C’, and D’), and a backing/turning module 60 (B’’, C’’, and D’’). Each analysis was conducted on a randomly drawn sample of 100 module blocks for each of the modules. B–B’’) Histograms showing the distributions of speeds in each of the modules. Different colors and shades indicated the direction and speed range of the data in that region. C–C’’) Frequencies of posture curve size and directions centered at each segment. Each curve centered at each segment was binned based on the size and direction of size and the dorsal or ventral direction of the curve. The size categories are as follows: S: 0–0.5 rad, M: 0.5–1.5 rad, and L: >1.5 rad. D–D’’) Segment curvature dynamics within and among sampled module blocks. The curvature values of each segment were converted to radian values. Curvatures bending ventrally were given the positive sign, while curvatures bending dorsally were given the negative sign. For each sample module block, the fifth percentile (lowest), the median, and the 95th percentile (highest) curvature values were extracted. Then distributions among each category are plotted and shown together. The boxes represent the range of 50% of the data surrounding the medians, which are represented by the lines in the boxes. The whiskers represent the range of 5–95 percentile.

Through the modeling, nine major behavioral modules were identified. One of them, module 1, is associated with failed data frames (Fig. S2B and C). Other modules are associated with locomotion, backing/turning, prodding, or the combination of the two or three (Fig. 4A). Locomotion is defined as the male moving not in contact with a hermaphrodite, which can be either forward or backward. Backing/turning and spicule prodding on the other hand involve the male interacting with the hermaphrodite and are part of mating. Locomotion and backing/turning each have one module associated uniquely with them, but spicule prodding has five different modules. This suggests that compared with locomotion and backing/turning, prodding behavior has various distinguishable subtypes used under different scenarios.

Consistent with the variability in mating latency, the usage frequencies are variable in both wild-type and hypo-ser males (Figs. 1C, 1D, and 4A). However, on average, wild-type males spent more time in locomotion and backing/turning module 9 and prodding module 23, while hypo-ser males spent more time in locomotion module 14 and prodding module 10. This result further supports that the variability in the behavior requires a library of animals to observe the distribution or compare different genotypes.

To further understand how each module is different, we conducted several statistical analyses and compared them among three modules 14, 60, and 40, each uniquely associated with locomotion, backing/turning, and prodding, respectively. First, we quantified the locomotion direction and speed of each module. As expected, locomotion module 14 is mostly moving forward, while backing/turning module 60 is mostly moving backward (Fig. 4B and B’’). Both of them had a preferred speed of 4 pixels/0.1 s (∼104 μm/s), but there are module blocks that can move in doubled speed or much slower in either forward or backward directions. On the other hand, prodding module 40 is essentially not moving with 98% of the blocks moving <1 pixel/0.1 s (∼26 μm/s; Fig. 4B’).

We next looked at how posture and its dynamics are defined in those modules. To understand the posture signatures associated with the modules, we converted the segment angle data into curves and quantified the sizes and positions of those curves (Fig. 4C–C’’). Since module 14 is a forward locomotion module, medium-sized (0.5–1.5 rad) and large-sized (>1.5 rad) ventral or dorsal curves were frequently found in most segments (Fig. 4C). Similarly, the backing/turning module 60 also showed medium- to large-sized curves in most segments, as the males propagated their body curvatures forward (Figs. 2B, 2C, and 4C’’). Interestingly, there is an obvious asymmetry in that more large ventral curves than large dorsal curves were found in the middle to posterior segments. We believe this is the result of the males exerting force to maintain their tail contact with the hermaphrodites during this module. The prodding module 40 however is showing a drastically different curve pattern from either module 14 or 60. Only ventral medium to large curves were observed in most of the segments. Occasional dorsal curves were seen in the anterior and middle segments. Only the tail region was showing either small or medium dorsal curves (Fig. 4C’). We believe this represents the stereotype prodding posture where the male slightly dorsally bends its tail and ventrally bends other segments to maintain ventral tail pressure during this mating step.

Finally, we examined the dynamics of postures both within each module block, as well as among different instances of the same modules. Since the module block sizes can be anywhere from 1 frame (0.1 s) to >100 frames (10 s), we took a sample of 100 module blocks with >10 frames to analyze the dynamics. To assess the degree of dynamics within a module block, we quantified the 5 percentile (lowest), median, and 95 percentile (highest) values of the segment angles, which represent what we call in-block variance. On the other hand, the distribution of those values among the module blocks gave us insight into the variability of the postures among the blocks or among-blocks variance. As expected, both forward locomotion module 14 and backing/turning module 60 have high in-block variance in all segments due to the constant changing of postures in these modules. On the other hand, the among-blocks variances contain fewer overlaps of the lowest, median, and highest distributions (Fig. 4D and D’’). This is because each segment goes through all degrees of bending during those moving modules. The overall posterior segments of module 60 showed a larger variance in the among-blocks variance and higher overlap compared with module 14. This is consistent with the observation that during backing/turning, the male tail is in close contact with the hermaphrodite, which confines the posterior end's range of motion, while the anterior body is generating waves to propel the male backward (Fig. 2B and C). Similar to other metrics, the posture dynamics of the prodding module 40 is drastically different from the other two modules. First, there is little in-block variance as the distributions of the lowest, median, and highest almost completely overlap with each other (Fig. 4D’). This is expected as the male barely moves during this module (Fig. 4B’). Interestingly, the among-blocks variance is not uniform across the segments. More specifically, middle-posterior segments 14–17 had a very small among-blocks variance compared with other segments (Fig. 4D’). This coincides with the prevalent medium to large ventral curves in that region (Fig. 4C’). These results suggest while other segments can be relatively variable, the middle-posterior segments are likely required to stay in a specific ventral bent status, providing the clamping force to maintain the tail's contact with the hermaphrodite's vulva during this prodding module.

### Maintaining vulva position during spicule prodding correlates with two differentially used modules

**Fig. 5.**
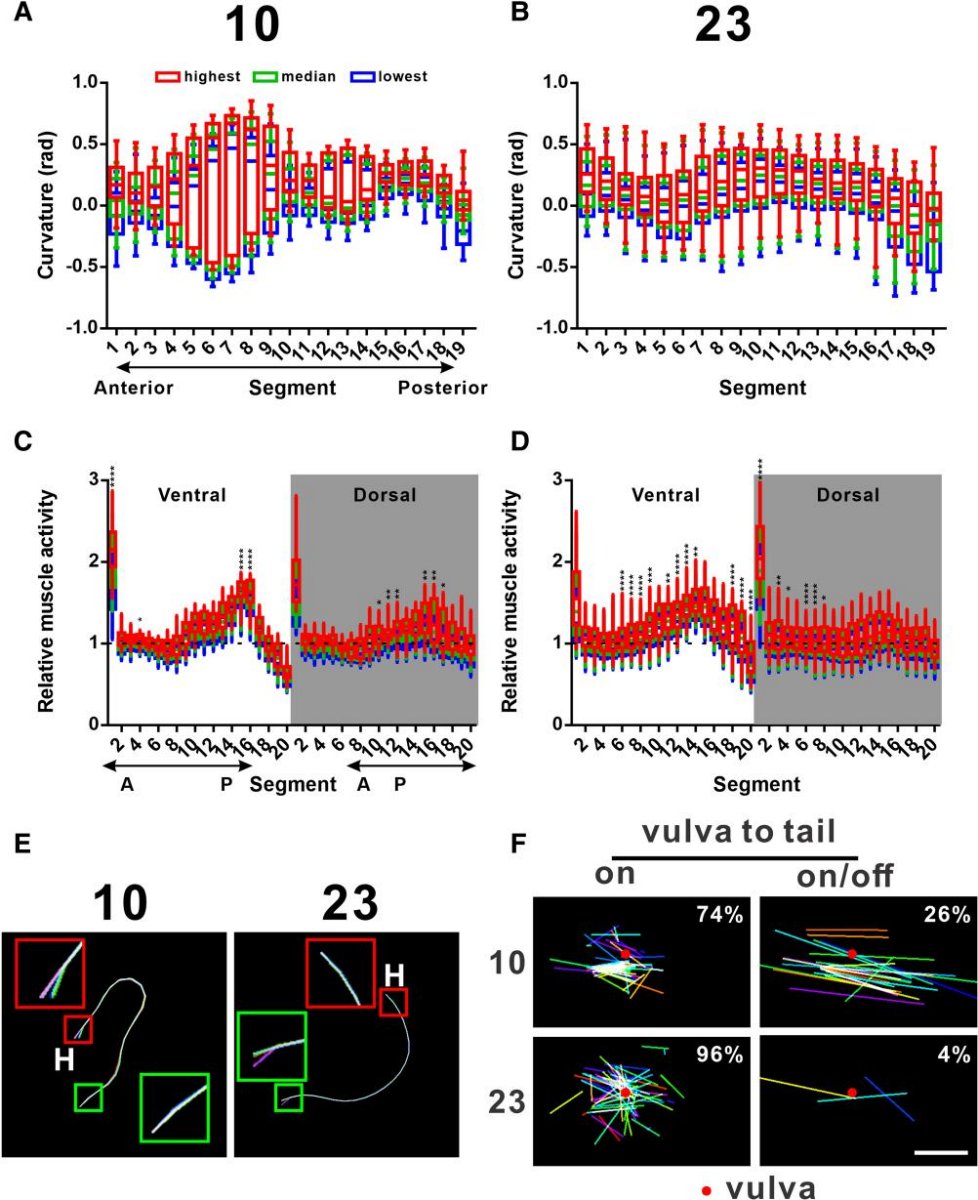
Comparative analysis of two distinct prodding modules. Segment curvature dynamics within and among sampled module blocks of module 10 (A) and module 23 (B). The curvature values of each segment were converted to radian values. Curvatures bending ventrally were given the positive sign, while curvatures bending dorsally were given the negative sign. For each sample module block, the 5th percentile (lowest), the median, and the 95th percentile (highest) curvature values were extracted. Then, distributions among each category are plotted and shown together. The boxes represent the range of 50% of the data surrounding the medians, which are represented by the lines in the boxes. The whiskers represent the range of 5–95 percentile. Segment muscle activity dynamics within and among sampled module blocks of module 10 (C) and module 23 (D). The distributions of the relative muscle activities were plotted to show the segment muscle activity dynamics. For each sample module block, the fifth percentile (lowest), the median, and the 95th percentile (highest) values were extracted. Then, distributions among each category are plotted and shown together. The boxes represent the range of 50% of the data surrounding the medians, which are represented by the lines in the boxes. The whiskers represent the range of 5–95 percentile. E) Overlapped skeleton graphs of prodding modules 10 and 23. One randomly selected module block was used to generate each graph. For each frame, the males' postures during these module blocks were reconstructed and graphed as a fixed-length skeleton curve representing the central axis of the male. Then, the curves from different frames were differentially colored and overlapped based on the center point of the animal. Regions with significant posture change would show as colorful branches or bundles, while regions with little posture change would show as white lines as all colors overlap. Small red and green boxes highlight the head and tail regions of the male, respectively. The bigger boxes are magnified images of their corresponding regions. H, head. A–D and F) Each analysis was conducted on a randomly drawn sample of 100 module blocks for each of the modules. F) Tail end positions relative to the vulva. The samples were divided into two panels: if the tails were continuously on the vulva (ON) or slipped off every now and then (ON/OFF). Each colored line represents the estimated range of positions of the tail relative to the vulva positions. The percentages on the corner indicate the proportion of module blocks in that category. Scale bar: 50 μm.

One of the surprising results is that five different prodding modules were found by the model (Fig. 4A). Previous studies have not identified any sub-types of prodding behavior, partially because apart from the contact between the tail and the vulva and the repetitive thrust of spicules, other potential characteristics of the behavior are difficult to describe in simple language. In our model, the algorithm not only found various spicule prodding modules, but it also identified a pair of prodding modules that were differentially used by wild-type and hypo-ser males. Module 23 was significantly used more frequently by wild type, while module 10 was almost exclusively used by hypo-ser males (Fig. 4A). To understand the significance of such differences, we looked closer at the differences between these two modules. We first applied a similar analysis that we used to examine the posture dynamics of the two modules. As expected, there was little in-block variance of all segments signified by the extensive overlap of the lowest, median, and highest distributions. However, we noticed drastic differences in the among-blocks variance. Specifically, module 10 had unexpectedly high among-blocks variance in segments 4–9 and to a lesser extent in segments 12–14. In other segments, the among-blocks variance was obviously smaller in module 10 compared with module 23 (Fig. 5A and B). A closer look at the distributions revealed that module 10 is bimodal at these two locations: the males are either heavily ventrally bent around segment 8 coupled with slight dorsal bending around segment 13 or heavily dorsally bent around segment 7 coupled with slight ventral bending around segment 13 (Figs. 5E and S4). This coordinated bending between segments seems to be one of the defining features of module 10.

We also looked at the distributions of muscle activities of these two modules. Mirroring what we observed in posture, the muscle activities in module 10 were much less variable among-blocks in several segments. Interestingly, even though segment 7/8 was extremely ventrally or dorsally bent in module 10, neither ventral nor dorsal muscle activity showed drastic among-blocks variance. When we focus on the in-block median level of the muscle activity, module 10 showed significantly higher median muscle activity in ventral segments 1, 15, and 16 and dorsal segments 10–12 and 15–17 than module 23. On the other hand, module 23 was much higher in ventral segments 6–9, 11–14, and 18–20 and dorsal segments 1, 3–4, and 6–8 (Fig. 5C and D). We believe this is evidence for differential coordinated muscle usage in these modules.

How did the different posture and muscle activity patterns affect the behavioral outcome of these two prodding modules? Were the males adjusting and fine-tuning their positions for successful prodding? To answer this question, we looked closer at the overall posture differences at whole-body level. We reconstructed the male postures during these module blocks and overlaid them. This highlights the regions that were changing during the behavior. Surprisingly, in the samples that we examined, males were changing their head segments' posture only in module 10 while changing their tail segments' posture only in module 23 (Fig. 5E). We hypothesize the different muscle usage coupled with the different posture resulted in different body parts adjusted during module 10 or 23. To see if these adjustments affect the prodding performance, we quantified the tail positions relative to the vulva during those module blocks. We found out that while the majority of the males were able to stay on the vulva all the time during these modules, significantly more incidences of off-vulva events occurred in module 10 (Fig. 5F). This suggests module 23 may be better at controlling the position of the tail to maintain the vulva contact during prodding than module 10. The significance of the body posture on spicule prodding, observed in module 10, requires further experimental dissection.

### Males execute mating behavior by repeating and transiting among bi-module blocks

**Fig. 6.**
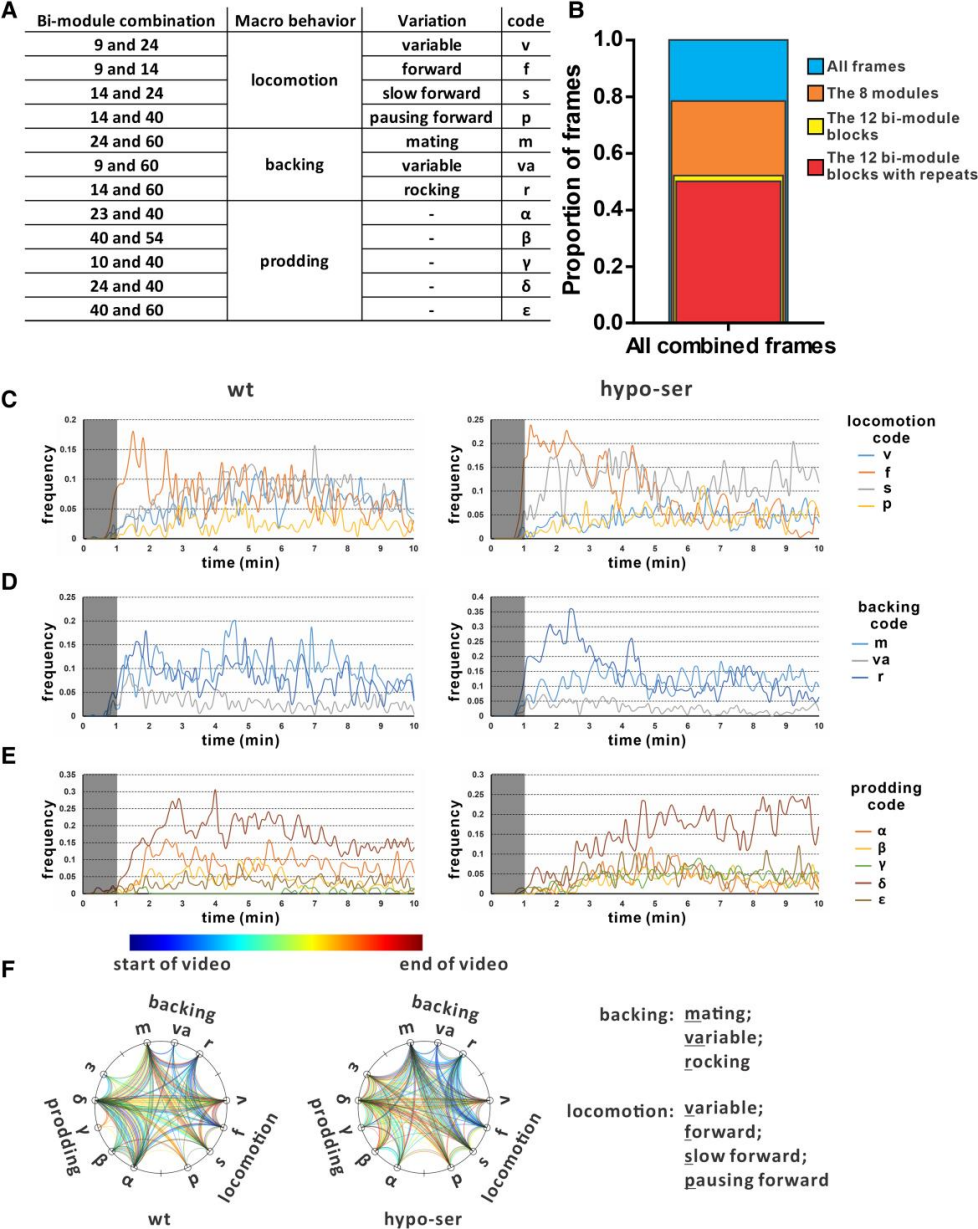
Bi-module repeats as the basic structure of mating behavior. A) A table summary of the 12 major bi-module combinations. B) The proportion of major modules and bi-module combinations or repeats in the data. C–E) Usage frequency of bi-module repeats over time in wild-type and hypo-ser males. Bi-module repeats were grouped based on the behaviors associated: C) locomotion, D) backing, and E) prodding. The gray shadows cover the first minute when most of the males had not been captured. Therefore, the frequency is not reliable during that timeframe. F) Aggregated path maps of wild-type and hypo-ser males. Each bi-module repeat version is a node on the circle. A colored line indicated a transition between the two connected bi-module repeats. All wild-type and hypo-ser data were aggregated separately to show patterns of paths among the bi-module repeats.

Even though we identified multiple behavioral modules that make up the male mating behavior, we still do not know how these modules were selected and organized for a successful mating event. We therefore examined the transition among the modules during the behavior. We found out that only 12 module pairs from the 8 major modules form bi-module blocks, representing the majority of the frames in the recordings. Even more surprising, these bi-module blocks almost always repeat when they are used. After examining the recordings of those bi-module repeats, we found four of them were involved in locomotion, three in backing, and five in prodding. We were also able to find and list some stereotypical differences among the various bi-module repeats of the same groups. For example, in locomotion, males were mostly continuously moving forward at a constant speed when they were in 9–14 (variation forward, coded as f) bi-module repeats. But, they would move much slower or even pause during 14–24 (variation slow, coded as s) or 14–40 (variation pause, coded as p) bi-module repeats. Similarly, backing also has different versions: the typical steady continuous backing on hermaphrodite during mating in 24–60 (variation mating, coded as m) bi-module repeats; the more hesitant, back and forth “rocking” version seen in 14–60 (variation rocking, coded as r) bi-module repeats or a more variable type in 9–60 (variation variable, coded as va). However, we could not easily identify unique features among the different versions of the prodding behavior, so we instead coded them using Greek letters to distinguish them (Fig. 6A and B). We hypothesize that while the patterns in the modules are generated by the activation of specific neuromuscular circuits, bi-module repeats form longer behavioral sequences by altered and repeated activation of two sets of neuromuscular circuits. This allows the males to complete certain behavioral steps when the length of behavior is nondeterministic. Under this definition, the bi-module repeats are the routines the males use to try and execute the multiple mini-goals in the mating behavior by a strategy we termed as “repeat until success/give up.”

To understand the function of the different bi-module repeats within the same behavior, we asked when those repeats were used in wild-type and hypo-ser males. In both cases, the f, continuous forward version locomotion, was mainly used at the beginning of the recordings as the males were navigating on the mating arena, searching for the hermaphrodites. However, the usage decreased in wild type after 2–3 min. Instead, the s, slow forward version locomotion, and the v, variable version (i.e. the behavior exhibited is too variable to define under this version) locomotion, were increasingly used at the same time (Fig. 6C). Since the majority of the wild-type males started mating before 3 min, the s and v versions locomotion are either associated with mating or shifted from the f version when males failed to locate hermaphrodite for a long time (Fig. 1C). For hypo-ser males, the f version usage decreased around 5 min, consistent with their slower start in mating compared with wild type (Figs. 1C and 6C). Interestingly, the s version was used more frequently and earlier, while the v version was consistently less used compared with wild type, clearly demonstrating the change of locomotion behavior by suppression of serotonergic neurons.

For the backing behavior, the mating-related m version was increasingly used as the f version locomotion usage decrease in wild type. Similar trend was also observed in hypo-ser males, but the overall usage frequency of the m version is lower than wild type. On the other hand, the r version, in which the males are hesitantly rocking back and forth, was much more used than wild type, especially during the first 4 min (Fig. 6D). Again, this divergence highlights the change in backing behavior in the hypo-ser males.

We showed that the model revealed five major prodding modules (Fig. 4A). Coincidentally, there were also five bi-module repeats associated with prodding, all of them involving the prodding module 40 (Fig. 6A). Among the five versions, the δ version was the most used prodding version in both wild-type and hypo-ser males. However, module 23-associated α version was the second most used prodding version in wild type, but the usage was much lower in hypo-ser males. At the same time, module 10-associated γ version usage was elevated in hypo-ser males, while in wild type, this version was only used sporadically after 6 min (Fig. 6E). These results further illustrate how hypo-ser males altered their prodding behavior by adopting different prodding versions and using less-used prodding modules.

To understand why these bi-module repeats were differentially used, we analyzed how males switched between the bi-module repeats. We plotted the switches by drawing curves connecting the bi-repeats. The curves were color coded to show when the switches occurred. The color and the thickness of the bundle can inform us of the timing and frequency of the specific path chosen by the males (Fig. 6F). Wild-type and hypo-ser males showed distinct path and temporal preference when they were switching among locomotion, backing, and prodding behaviors. For wild-type males, early transitions occurred between the f version locomotion and the r version backing (f–r connection), suggesting the typical entry point for mating behavior. In hypo-ser males, the same path was also used frequently between locomotion and backing. But they also had more early connections between the f version locomotion and the va version backing (f–va connection) and between the s version locomotion and the r version backing (s–r connection). Also, extra connections between the p version locomotion and the m version backing (p–m connection) as well as the earlier shift between the s version locomotion and the m version backing (s–m connection) provide extra routes between locomotion and backing. Combining this result with the fact that hypo-ser males on average took longer to start mating, we hypothesize hypo-ser males use those connections as alternative paths for mating entry (Figs. 1C, 1D, and 6F).

Pertaining to transits related to the prodding behaviors, in both wild-type and hypo-ser males, the δ version of prodding was the major hub connecting other behaviors to prodding and mediating transitions among different prodding versions. Between backing and prodding, the major path was between the m version backing and the δ version prodding (m–δ connection). The δ version prodding was also frequently connected to the v version locomotion (v–δ connection). Considering that the occurrence of these transitions occurs late into the recordings, we believe this connection may represent when the males exit the mating behavior. On the other end, the transitions among the prodding versions almost always connected to the δ version prodding as well. While the wild-type males switched between δ–α and δ–β during prodding, interestingly, the hypo-ser males had less transitions between δ–α with increased δ–γ transitions at the same time. This altered preference in transition explains the elevated usage of γ version prodding and the decreased usage of α version prodding (Fig. 6E and F).

## Discussion

The mating behavior of *C. elegans* males is the most complex behavior exhibited by this animal with only 387 neurons (3, 32, 43). We demonstrated a method to dissect a behavior that is highly variable and with elements that are difficult to describe. By focusing on the muscles, we were able to generate a library of >100 animals just by manually tracking the animals under a low magnification 4× objective. As an added bonus, we kept posture information when we recorded the whole animal instead of just the brain region (Fig. 2A). Advancement in microscopy allowed tracking of a population of *C. elegans* neurons while the animal is performing behaviors including mating (25, 30). However, such technology is still in its infancy and challenging to scale due to the difficulty in tracking the animal under high magnification and identifying the different neurons from the population. Even with multicolor labeling of neurons and partial-automated labeling, such studies were only able to analyze 10 or less animals with enough quality datasets (27, 29). Efforts have been made to use artificial intelligence to aid the tracking and identification of neurons, but its ability and usefulness are yet to be tested (44). Using our method and fully automatic analytic package, NAWA, we can automatically process the recordings and extract the relevant posture and muscle activity data, increasing the throughput of the analysis.

To demonstrate the scalability and robustness of this system, we did a benchmark experiment upon the completion of this paper. We ablated HOA + HOB neurons in wild-type males carrying our transgene and made 11 mating recordings with those males. We then analyzed the recordings using NAWA and the AR-HMM model. Just one trained researcher can finish the whole process in less than a month. Since HOA and HOB are essential for males to locate the vulva, the males had difficulty in stopping on the vulva and prodding on the vulva. Analysis of the results showed a reduced use of prodding modules and an increased use of backing modules, consistent with the function of the neurons (Fig. S5). We also drafted a step-by-step manual on how to prepare and process recordings using NAWA, which should help anyone interested to implement NAWA in their research (45).

In this system, we evenly segmented the males into 20 segments, with each segment roughly corresponding to 1 set of the 4 body wall muscles in quadrants (31, 33). Therefore, each ventral or dorsal segment roughly contains two overlapped body wall muscles. A more detailed dataset at individual cell level especially at the tail region can be informative since many small muscles are there (Fig. 1B). However, due to the constraint of the resolution of the microscope and camera, we are approaching the accuracy limit when we divided the worm into 20 segments. Right now, each segment is only about 10–20 pixels long. Further dividing or having denser regions will cause the segments' quantification to be volatile to noises and inconsistencies among frames. However, if the microscope and camera setup improve in the future, we will certainly attempt to segment the worm in finer detail. For now, the 20 segments still give us close to single-cell resolution for the body wall muscles since the two quadrants on each of the ventral/dorsal side are essentially overlapped and their contractions are synchronized.

In our AR-HMM model of the mating behavior using the posture and muscle activity data, we provided a hierarchical description of the behavior. Those units should be reused by different animals and represent a programmed activation of specific circuits. We selected AR-HMM specifically because it captures the hierarchical structure of the behavior. The autoregressive model captures the inner dynamics of each programmed module, while the Markov model represents the probabilistic nature of the decision-making process (Fig. 3). The same model was used in understanding mice behavior with posture data from a single 3D camera (42). We successfully identified five major prodding modules in addition to the common forward locomotion and backing/turning modules. More importantly, the different posture and muscle activity dynamics were clearly demonstrated along with their performance in maintaining vulva contact during prodding (Fig. 5). Because of this modular approach, we were able to discover the prevalent bi-module repeat structure in the behavior (Fig. 6). This may be the manifestation of the decision-making strategy of the males to achieve a multistep goal with undefined time requirement and difficulty for the smaller goals.

Many studies modeled neuronal dynamics. Similarly, they dissect the behavior into multiple states and fit the data using models such as switching linear dynamical systems (26). Those efforts showed promise in revealing the control signals and predicting the dynamics of the behavior. However, because of the limited number of animals and the constrain of the behaviors exhibited in the dataset, only few simple known behaviors such as forward locomotion, reverse locomotion, ventral turn, and dorsal turn were explained by the models (46, 47). Even though our approach lacks a direct understanding of the neuronal dynamics behind the posture and muscle activity data, we can potentially model the neuronal activities from these data. Studies in monkeys successfully predicted neuronal activity in the motor cortex just by modeling the movement in an artificial neuron network (48, 49). Given the more detailed information we have in the *C. elegans* connectome and actual calcium activity from the muscles, there is great hope that we can reconstruct the decision-making process of the animal.

Since wild-type males showed variability in their mating behavior, we decided to include a strain with perturbed neural excitability, challenging our system to distinguish the difference. Serotonin is one of the biogenic amine neurotransmitters that control behaviors such as egg laying and pharyngeal muscle pumping in *C. elegans*. It can also be released as a hormone to coordinate general behaviors such as locomotion with environmental cues (50, 51). In addition to the sex-shared neurons NSM, ADF, AIM, and RIH, serotonin is made in several male-specific neurons including three pairs of type B ray sensory neurons 1, 3, and 9 and the ventral motor/inter posterior cord 1–6 neurons (52–54). A study from the last century showed serotonin-deficient males had defects in turning behavior, and serotonin can induce tail curling (7). Since then, only a few studies examined the function of these serotonergic neurons in tail curling or mate-searching behaviors. We believe those neurons have extra and potentially more complex functions in regulating behaviors. Since most of the serotonergic neurons express more than one neurotransmitter, mutating serotonin production enzymes would not attenuate those neurons. Instead, we used *tph-1* promoter to drive a gain of function potassium channel to tune down the overall activity of all serotonergic neurons, making the hypo-ser males. This approach also allows us to study any other neurons using the same method by replacing the promoter, simplifying and standardizing the process. Using this method and comparing the data with wild-type males, we found hypo-ser males took longer to mate, consistent with the reduced food-leaving mate-searching behavior shown in serotonin-deficient males (Fig. 1C and D) (55). But when we applied NAWA and the model, we were able to statistically quantify the differential behavioral module usage by the hypo-ser males (Fig. 4A). More specifically, we identified a prodding module that was significantly used more frequently in hypo-ser males at the expense of reducing the use of another prodding module. These modules showed differences in vulva contact maintaining performance (Fig. 5F). It would be interesting to dissect the neuromuscular circuit difference that generated such changes.

*C. elegans* male mating behavior is the most complex behavior exhibited by this organism. Still, its complexity may still be underestimated with many more features of this behavior yet to be discovered. Recently, a previously-unknown stereotyped movement during mating termed “Molina maneuver” was discovered. This happens when the male has not been successful in inserting spicules and losing vulva apposition. The male may exhibit this movement by locomoting forward for at least two tail tip lengths followed by a deeply arched posture in the tail and backing to relocate the vulva (43). While we did not identify a specific module that matches this movement, it is possible that sub-behaviors like this are made up of a combination of modules described in this work. We believe our system has the great potential in complete dissection of the behavior. NAWA is not bound by specific posture, so it can be used to analyze males with different genetic backgrounds or conditions. Similarly, for researchers interested in different questions, we can just retrain the system to use it on a different animal carrying a different fluorescent reporter without modifying the downstream analysis. This would be trivial as it only required labeling of ∼6,000 randomly picked video frames to train the current system (Supplementary Methods). Thus, a different behavior or different sub-systems of the organism can be easily adapted for this system.

## Materials and methods

See Supplementary Methods.

## Supplementary Material

pgae032_Supplementary_Data

## Data Availability

All original data and code created for the study are linked and available for download from Harvard Dataverse along with a step-by-step instruction on how to use the code (45).
