# Supplementary material for: Unraveling the hierarchical structure of posture and muscle activity changes during mating of Caenorhabditis elegans
Source: PNAS Nexus. 2024 Jan 24;3(2):pgae032. doi: 10.1093/pnasnexus/pgae032 (PMC10837012; doi:10.1093/pnasnexus/pgae032)
Supplement: pgae032_Supplementary_Data [file pgae032_supplementary_data.docx]

**
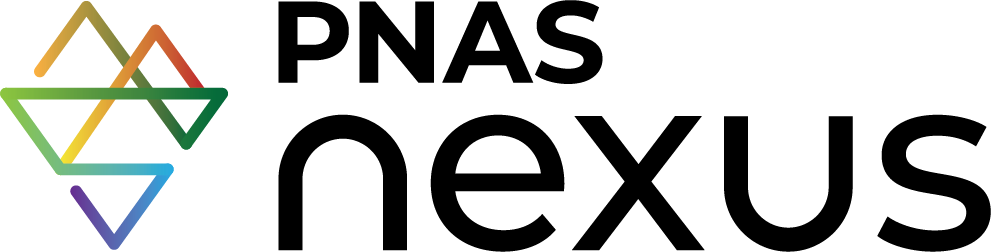
**

**Supplementary Information for**

Unraveling the hierarchical structure of posture and muscle activity changes during mating of *C. elegans*.

Yufeng Wan, Luca Henze Macias, Luis Rene Garcia

Department of Biology, Texas A&M University, College Station, TX, 77843

* Luis Rene Garcia

Email: rgarcia@bio.tamu.edu

**This PDF file includes:**

Supplementary text

Figures S1 to S4

SI References

**Supplementary Information Text**

**Supplemental Methods.**

***Strains***

Nematodes were cultured at 20 °C on NGM agar plates with E. coli strain OP50 as food source (1), except strain with *pha-1*(*e2123*), which was maintained at 15 °C (2). The alleles used were: *lite-1*(*ce314*) (3) on LGX; *pha-1*(*e2123*), *unc-64*(*e240*) (1), on LGIII, *him-5*(*e1490*) (4) on LGV.

***Plasmids and transgenic strains***

To generate body wall and sex muscles expressing GCaMP plasmid pBL340, *lev-11* promoter from pLR22 (5) was recombined with GCaMP6:SL2::mDsRed containing Gateway plasmid pLR305 (6) using LR clonase (Invitrogen). A mixture of 10 ng/µl pBL340, 25 ng/µl pPD132.102 [P*myo-2*:GFP] (Addgene) and 165 ng/µl pUC18 was then micro-injected into N2. After a stable transgenic line was obtained, the extrachromosomal array was integrated into the genome using trimethylpsoralen and UV to promote DNA breakage and repair (7). The extrachromosomal array integrated into chromosome II to generate the transgenic allele rgIs24.The strain was further crossed to generate *lite-1; him-5;* rgIs24.

To generate vulva expressing GCaMP plasmid pLR289, *unc-103E* promoter from pLR21 (8) was recombined with G-CaMP3:SL2::mDsRed containing Gateway plasmid pLR279 (9). A mixture of 20 ng/µl pLR289, 50 ng/µl *pha-1*(+) rescuing plasmid pBX1 (10), and 130 ng/µl pUC18 was then micro-injected into *unc-64*; *pha-1*; *him-5* to generate extrachromosomal array rgEx567.

To make *unc-103(gf)*:SL2::mDsRed containing Gateway plasmid pMM1, SL2::mDsRed from pLR279 (9) digested with *BamH*I and *Not*I was ligated with *unc-103(gf)* containing Gateway plasmid pLR73 (11) digested with *Not*I and *Spe*I. *tph-*1 promoter from pTG9 (12) was then recombined with pMM1 to make serotonergic neurons expressing unc-103(gf):SL2::mDsRed plasmid pYW65. A mixture of 20 ng/µl pYW65, 50 ng/µl *pha-1*(+) rescuing plasmid pBX1, and 130 ng/µl pUC18 was then micro-injected into *pha-1*; *lite-1;* *him-5* to generate extrachromosomal array rgEx888.

***Mating recording collection***

*Mating plate*

To reduce the auto fluorescence of NGM plates, we made mating plates by dissolving 3% BD DIFCO™ Noble Agar in S Medium (13) and pouring into a 60x15 mm petri dish. 10 µl of concentrated OP50 was spotted on the mating plate and let dry as the mating lawn.

*Strain preparation*

To limit the effect of GCaMP on male’s ability to mate, *lite-1*; *him-5*; *rgIs24* males were crossed with *pha-1*; *lite-1*; *him-5* hermaphrodites to produce heterozygous cross-progeny for wild type and HOA+HOB ablated datasets. *pha-1*; *lite-1*; *him-5*; rgEx888 males were crossed with *pha-1*; *lite-1*; *him-5*; rgIs24 hermaphrodites to produce heterozygous cross-progeny for hypo-ser datasets. This results in reduced expression of the transgene. Cross-progeny males at L4 stage were transferred to a new plate with E. coli OP50 lawn and left to mature overnight. As their mates, L4 hermaphrodites of *unc-64*; *him-5*; rgEx567 were transferred to new plates to mature overnight. In total 62 wild type and 63 hypo-ser males were used in making the recordings.

*Laser ablation*

To ablate HOA and HOB sensory neurons, precursor cells P9.p and P10.p were ablated (using a Spectra-Physics VSL-337ND-S nitrogen laser attached to an Olympus BX51 microscope via the MicroPoint laser focusing system) in L2 or L3 males. During the operation, the males were immobilized between a microscope coverslip and a 3% noble agar pad containing 3mM of NaN_3_ and Polybead polystyrene 0.1 µm microspheres (Polysciences, Inc., WA). 24 hrs latter, these males were used for recordings.

*Noble Agar cover slip*

To improve optics of the imaging without disturbing mating, lightweight and soft cover slips were made from 0.8% Noble Agar. To make the agar cover slips, two strips of labeling tape were taped on a microscope slide about 2.5 cm apart, serving as the spacers. One piece of plastic wrap cut in 2x4 cm was then wrapped over the microscope slide between the spacers as the base. Another same sized plastic wrap was wrapped over an empty microscope slide. 20 µl of 0.8% Noble Agar was then dropped on the base wrap. Another wrapped clean microscope slide was immediately flat dropped above the agar, resulting in a roughly agar pad in 6 mm diameter and 1 mm thickness. After 5 min of solidification, the slides were separated. The side with the agar can be peeled off with the plastic wrap and be used as a Nobel Agar cover slip.

*Mounting and recording*

For each mating, 10 adult hermaphrodites were picked and spaced circularly on the mating lawn of OP50. After the hermaphrodites acclimated and releasing their pheromones for one hour, the chunk containing the mating lawn and the hermaphrodites was then placed on a microscope slide. A male was placed in the center of the hermaphrodite ring followed by placing a Noble Agar cover slip over the chunk (**Supplementary Figure 1A**). The slide was immediately placed and recorded on a fluorescence-equipped Olympus BX51 microscope (Olympus, USA). The male was tracked and recorded manually under 4x objective for 10 min since on the pad or when it moved off the hermaphrodite, whichever happened first. The GCaMP and mDsRed channels were recorded simultaneously by the Dual View Simultaneous Imaging Systems with an OI-11-EM filter by Photometrics (Surrey, BC) and a Hamamatsu ImagEM Electron multiplier CCD camera, at the speed of 10 frames per second and a resolution of 512x512 pixels using the software HCImageLive (Hamamatsu Photonics K.K.) (**Supplementary Figure 1B**).

*Scripts and programs*

All lab-written/built scripts and programs used to analyze the recordings and model the data were written using MATLAB R2017a (Mathworks), including codes downloaded from the MATLAB community. Scatter plots, bar graphs, and graphs of module dynamic analysis were generated using GraphPad Prism.

*Recording pre-processing*

Recordings were trimmed to the first frame with a focused male and exported as multi-page 16-bit 512x512 TIFFs using HCImageLive. Then a lab-written script processed each image by dividing and aligning the Red (DsRed) and Green (GFP+GCaMP) channel then storing each channel picture in a new 16-bit 512x256 RGB TIFF image’s red and blue channels, respectively.

***NAWA analysis***

*Preparing training data for NAWA*

From each of the 62 wild type videos recorded, 100 random frames were selected. Using a lab-written script, each image was then annotated by a human to label the positions of the male’s head, tail, dorsal and ventral body walls, as well as the position of the hermaphrodite’s vulva. The script then generate 6 8-bit 512x256 grey scale images, each using the brightness of pixels to label the position of hermaphrodite vulva, head, tail, dorsal and ventral body walls, and the whole body of the male. From these 6200 annotated images, 5100 were randomly chosen to be training data and 1100 as testing data to verify the training quality.

*Constructing of NAWA*

NAWA system include 2 parts. First, to construct an artificial deep neural network (DNN), we modified a deep learning network FCN-8s from the open source library MatConvNet (14). In brief, we modified the input layer and output layer to fit our image size. We also modified the loss function to sigmoid cross entropy loss to generate probabilistic maps instead of classification maps. Since the ventral and dorsal body walls would switch their relative positions in relation to the AP axis, we instead asked artificial neural network to predict the “left body wall” defined as the ventral body wall when the male lies on its right or the dorsal body wall when the male lies on its left, and “right body wall” as the other side. We also used two extra DNNs to train the “left body wall” and the “right body wall” separately from the other parts because they are significantly more difficult to train than the others. We also used codes from MatConvNet for the training and inference of the DNNs. During the training process, the parameters in the DNNs were refined to match the predictions with the annotations. After training, the trained DNNs were then used to infer all the datasets to produce 8-bit 512x-256 grey scale images for each of the body parts in each frame. The training and inference was done on a desktop PC equipped with Intel I5 8400 and Nvidia GTX 1060 6 Gb.

The second part is an algorithm to reconstruct the skeleton of the males from the body parts labels predicted by the DNNs. The skeleton is represented by serious of points running from the head to the tail, representing the center line of the worm. First the algorithm locates the head and tail positions by finding the brightest pixels in the head image and the tail image processed with a Gaussian filter. Then the next position in the central line posterior to the head is found by examining 360 points surrounding the head point 10 pixels (about 26 μm) away. Then the likeliness of correctness is calculated using a series of energy functions inspired from protein structure modeling with higher energy correspond to less likeliness in correct position. This process is then repeated until reaching the tail of the worm.

The first energy function calculates the percent of not-whole body in local area of candidates. For each candidate point $\mathcal{i\in}\left\{ 1,2,\ldots,360 \right\}$ with coordinate$\left( X_{i},Y_{i} \right)$, matrix $R_{region}$ representing pixels in local area are found by the following test:

$$R_{region}=\sqrt{\left( \left( mat_{Y}-Y_{i} \right)^{2}+\left( {mat_{x}'-X}_{i} \right)^{2} \right)}\leq10$$

where $mat_{Y}=1:512$ and $mat_{x}=1:256$ are vectors representing the coordinates of all pixels in the images. The first energy is calculated by:

$$E_{1}≞\frac{\sum\left( 1-R_{body} \right)\odot R_{region}}{\sum R_{region}}$$

where $R_{body}$ is the normalized matrix from the predicted whole body grey scale image.

The second energy function calculates the distance of the body center in the local area of candidates to the candidate axis:

$$E_{2}≞\left| \frac{\left( Y_{vec}\times X_{cen}-Y_{cen}\times X_{vec}+Y_{pre}\times X_{i}-Y_{i}\times X_{pre} \right)}{10} \right|$$

where $\left( X_{pre},Y_{pre} \right)$ is the coordinate of previous known point in the center line and $\left( X_{cen},Y_{cen} \right)$ is the coordinate of the body center in the local area found by:

$$X_{cen}=\frac{\sum\left( R_{body}\odot R_{region}\odot mat_{x}' \right)}{\sum R_{region}}$$

$$Y_{cen}=\frac{\sum\left( R_{body}\odot R_{region}\odot mat_{Y} \right)}{\sum R_{region}}$$

$\left[ X_{vec},Y_{vec} \right]$ is the vector from $\left( X_{pre},Y_{pre} \right)$ to$\left( X_{i},Y_{i} \right)$.

The third energy function calculates the ratio between the body wall parts in the local area and the body wall parts in the correct side in the local area:

$$E_{3}≞\left. \frac{\left( 1+\sum\left( R_{left}\odot R_{region} \right) \right)}{\left( 1+\sum\left( R_{left}\odot R_{l\_region} \right) \right)}+\frac{\left( 1+\sum\left( R_{righ}\odot R_{region} \right) \right)}{\left( 1+\sum\left( R_{righ}\odot R_{r\_region} \right) \right)} \right.$$

where $R_{left}$ is the normalized matrix from the predicted “left body wall” grey scale image and $R_{right}$ is the normalized matrix from the predicted “right body wall” grey scale image. Matrices $R_{l\_region}$ and $R_{r\_region}$ representing pixels in the left or the right part of the local area, respectively. They are found using the following test:

$$R_{l\_region}=\left( \left( mat_{Y\_vec}{\odot X}_{vec}-mat_{X\_vec}{\odot Y}_{vec} \right)>0 \right)\odot R_{region}$$

$$R_{r\_region}=\left( \left( mat_{Y\_vec}{\odot X}_{vec}-mat_{X\_vec}{\odot Y}_{vec} \right)<0 \right)\odot R_{region}$$

$$mat_{Y\_vec}=mat_{Y}-Y_{pre}$$

$$mat_{X\_vec}=mat_{X}-X_{pre}$$

The fourth energy function calculates the imbalance of the “left body wall” and the “right body wall” in the local region:

$$E_{4}≞\left| d_{left}-d_{right} \right|$$

$$d_{left}=\left| Y_{vec}\odot mat_{lx}-X_{vec}\odot mat_{ly}+X_{i}\times Y_{pre}-Y_{i}\times X_{pre} \right|⊘\sqrt{\left( {Y_{vec}}^{2}+{X_{vec}}^{2} \right)}$$

$$d_{right}=\left| Y_{vec}\odot mat_{rx}-X_{vec}\odot mat_{ry}+X_{i}\times Y_{pre}-Y_{i}\times X_{pre} \right|⊘\sqrt{\left( {Y_{vec}}^{2}+{X_{vec}}^{2} \right)}$$

$$mat_{lx}=\left. \sum\left( {mat_{x}'\odot R}_{l\_region}\odot R_{left} \right) \right.⊘Maximium(1,\sum\left( R_{l\_region}\odot R_{left} \right))$$

$$mat_{ly}=\left. \sum\left( {mat_{y}\odot R}_{l\_region}\odot R_{left} \right) \right.⊘Maximium(1,\sum\left( R_{l\_region}\odot R_{left} \right))$$

$$mat_{rx}=\left. \sum\left( {mat_{x}'\odot R}_{r\_region}\odot R_{right} \right) \right.⊘Maximium(1,\sum\left( R_{r\_region}\odot R_{right} \right))$$

$$mat_{ry}=\left. \sum\left( {mat_{y}\odot R}_{r\_region}\odot R_{right} \right) \right.⊘Maximium(1,\sum\left( R_{r\_region}\odot R_{right} \right))$$

The fifth energy function calculates the amount of change in the direction of the central axis:

$$E_{5}≞\left\{ \begin{aligned} 0; \left( X_{i},Y_{i} \right) is the first point after head \\ \left( -5\times\log{Cos}_{c} \right)^{5};\left( X_{i},Y_{i} \right) is the second point or later after head \end{aligned} \right.$$

$${Cos}_{c}= Maximium(0.1\times\left( \cos\theta_{i}\times X_{p\_vec}+\sin\theta_{i}\times Y_{p\_vec} \right),0)$$

$$\theta_{i}=\pi\times\frac{i}{180}$$

where $\left[ X_{p\_vec},Y_{p\_vec} \right]$ is the vector from two points before $\left( X_{i},Y_{i} \right)$, $\left( X_{p\_pre},Y_{p\_pre} \right)$ to$\left( X_{pre},Y_{pre} \right)$. The total energy is them calculated as:

$$E≞\sum_{k=1}^{5} w_{k}\times E_{k}$$

where the weight set $\left\{ w_{k} \right\}_{k=1}^{5}$ is empirically set as $\left\{ 4,5,10,1,1 \right\}$ by trial and error.

The next point in the axis is then found by searching the $\left( X_{i},Y_{i} \right)$ with the lowest total energy $E$. This process is then repeated until the tail is within 20 pixels away from the last point, in which point the tail will be added as the end point of the center line.

Finally, the algorithm divides the line into 20 even segments and extract the angle changes between adjacent segments and relative calcium activities of each side of the 20 segments represented by the ratio between the green and red channels in the region (**Figure 2A**).

*Data processing and quality control*

After the DNNs in NAWA were trained to satisfaction, all recordings were fed into NAWA to extract the posture and muscle activity data along with pictures showing the reconstructed worm skeletons. We manually scanned through the reconstructed worm skeletons to identify when the system failed to model the worm correctly and pruned the data in such cases (**Supplementary Figure 2C**). We also used linear interpolation to fill the short (less than 10 frames) missing data using neighbouring frames. Because the transgenic GCaMP fluorescence varies greatly from male to male even with integration, we normalized each segment’s calcium activity with the median Green to Red ratio of all segments throughout the recording for each worm.

*Model free analysis*

As a reference and a control for the noise in the system, we recorded 10 immobilized fluorescent wild type males each for 10 minutes and processed their data in addition to other data for the model free analysis.

The principal component analysis (PCA), autocorrelation, and the Welch’s power spectral density estimate were conducted using the built-in functions in MATLAB.

To estimate the approximate block sizes of behaviors, we used a changepoint identification system, which was previously used in a mice behavior study that automatically identify the boundaries between blocks (15). The algorithm is called the filtered derivative algorithm (16). The algorithm calculated the derivative of the measurement data or the 30 top principal components (PCs) with a lag of k frames. Then it binarized using a threshold h, summed all dimensions, resulting in a one-dimensional signal, which was smoothed with a Gaussian filter with a standard deviation of s frames. Changepoints were then identified as the local maxima of the smoothed one-dimensional signal. The values of the parameters k, h and s were identified by maximizing ratio between the number of changepoints in the behaving males and the immobilized males. The resulting parameter values are as follows: k=11, h=2.1, s=1.

*Data modeling and fitting*

The generative AR-HMM and the fitting process is extensively explained in another paper (15). Briefly, the data was first compressed by using the 30 top PCs for the analysis. Then the AR-HMM was fit using Gibbs sampling iteratively (17, 18). In this model, two parameters cannot be fitted and had to be set manually. First is the maximum time lag K frames in the autoregressive model. The maximum time lag determines the size of the autoregressive model. We chose K=30 frames based on the autocorrelation analysis showing the short-term dynamics of the data is shorter than 3 seconds (**Supplementary Figure 3B**). The next parameter is a sticky bias parameter kappa in the HMM. This parameter affects the duration distribution of modules by biasing the self-transitions of modules. We tested a series different kappa and selected a value of 2e+12 based on the best match between the resulting block size and the changepoint analysis (**Supplementary Figure 3D-E’’’**).

*Analysis of behavioral modules*

Lab-written scripts were used to isolate and rank the modules identified during the modeling process. The raw video recordings associated with the top modules were visually examined to associate the behavioral step with the modules. For quantitative analysis, 100 blocks with >1 second duration of each module were randomly selected.

The locomotion speed in each block was estimated by shifting the body segments at different distance anteriorly or posteriorly along the skeleton, comparing difference between the resulting posture with the actual measurement, finding the distance with the smallest difference and calculating the correspond speed.

To estimate the size of curves the male is showing, adjacent curvatures to the same side were joined to calculate the total ventral/dorsal curvature of a continuous series of segments.

To estimate the relative position of male tail to tail in the prodding modules 10 and 23, the same algorithm in NAWA used to identify the head and tail position was used to identify the vulva position in the sample blocks. Then the relative position of the male tail to vulva was reoriented using the last body segment of the male. To plot the tail to vulva position graph, the most anterior position and the most posterior position of the tail relative to the vulva were found, a line was then drawn connecting the two points signifying the range of tail positions of the tail to vulva. The distance between the two points was also calculated for classifying each block into on vulva, off vulva or on/off vulva. The criteria for on vulva include: both points are within 8.5 pixels (~22 μm) of vulva location; the distance of the range <11 pixels (~29 μm). The criteria for off vulva include: either points are outside 8.5 pixels of vulva location. The criteria for on/off vulva include: both points are within 8.5 pixels (~22 μm) of vulva location; the distance of the range >11 pixels (~29 μm).

*Analysis of bi-module repeats*

Lab-written scripts were used to identify and rank the bi-module repeats. The raw video recordings of associated with the bi-module repeats were visually examine to associate the behavioral versions with the bi-module repeats. The usage frequency and timing of the usage were quantified using lab-written scripts as well.


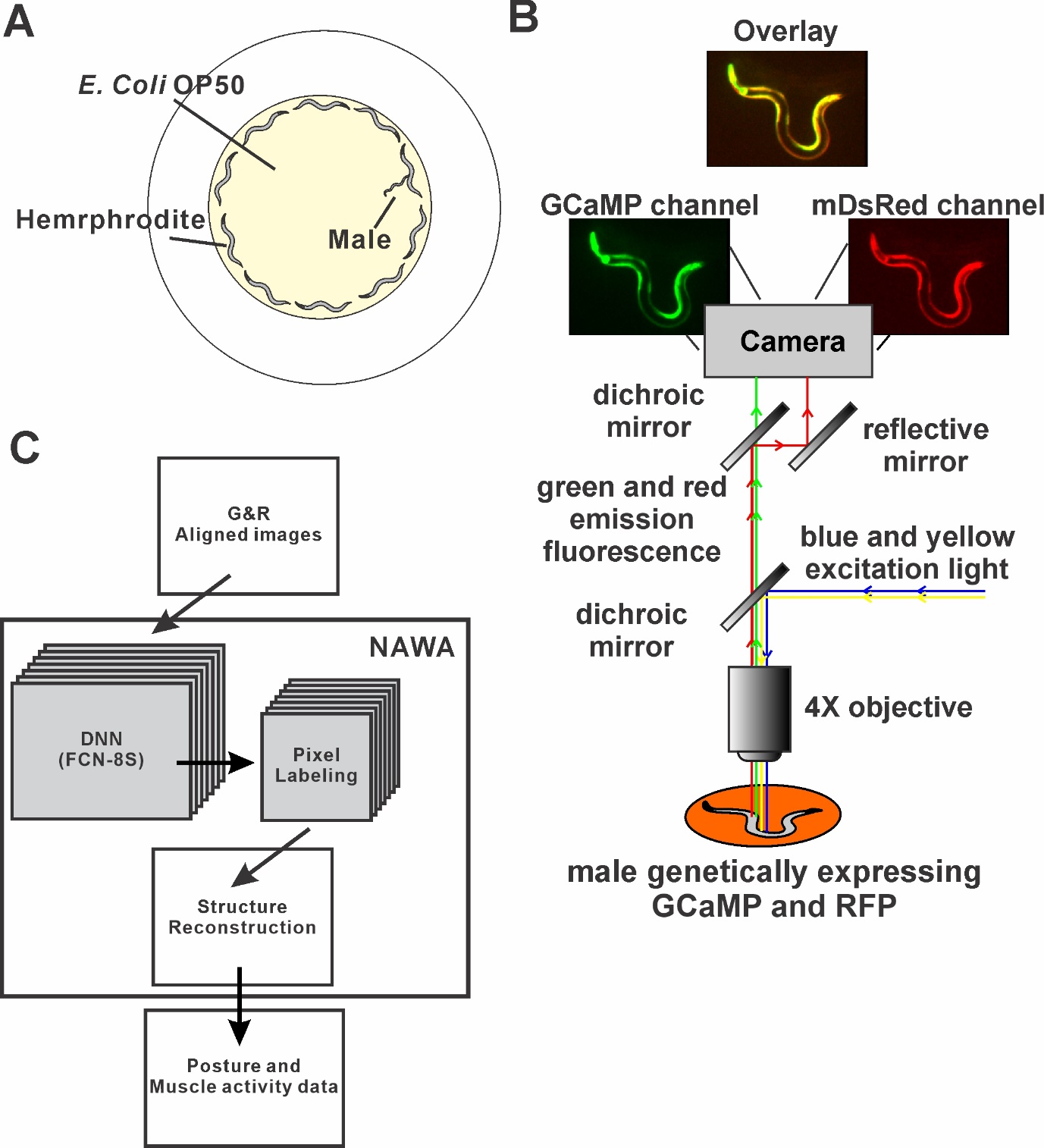


**Fig. S1.** A) Illustration of the mating arena. B) Schematic of light paths in the fluorescent microscope. C) Illustration of major components in NAWA.

**
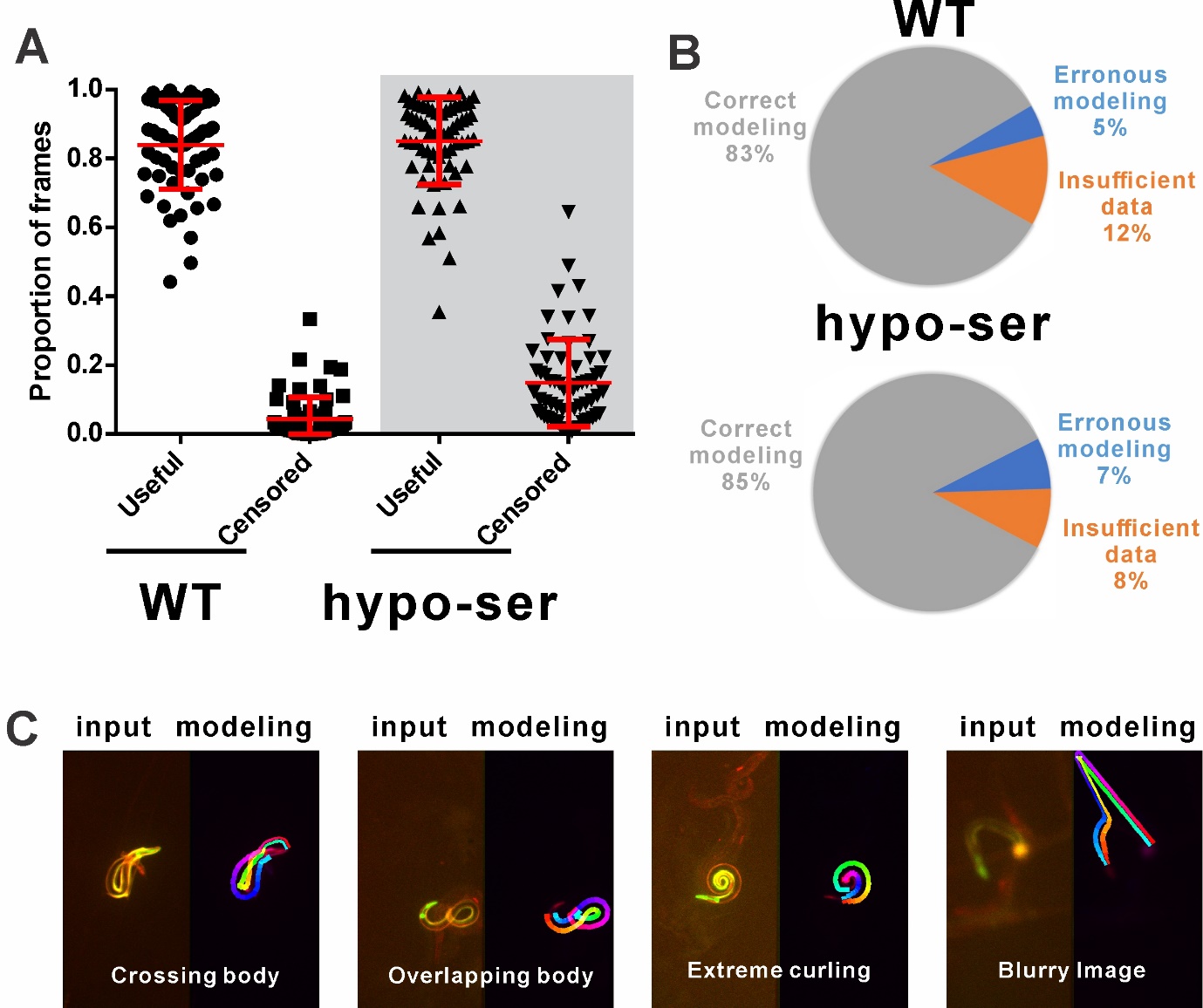
**

**Fig. S2.** A) Proportion of frames useful or censored in each of the wild type or hypo-ser male recordings. Each point represents one recording. Red bars and error bars represent mean±S.D. B) Pie chart breaking down of the total frames in the wild type and hypo-ser datasets. C). Example raw image frames and the modeling outputs of Erroneous modeling.

**
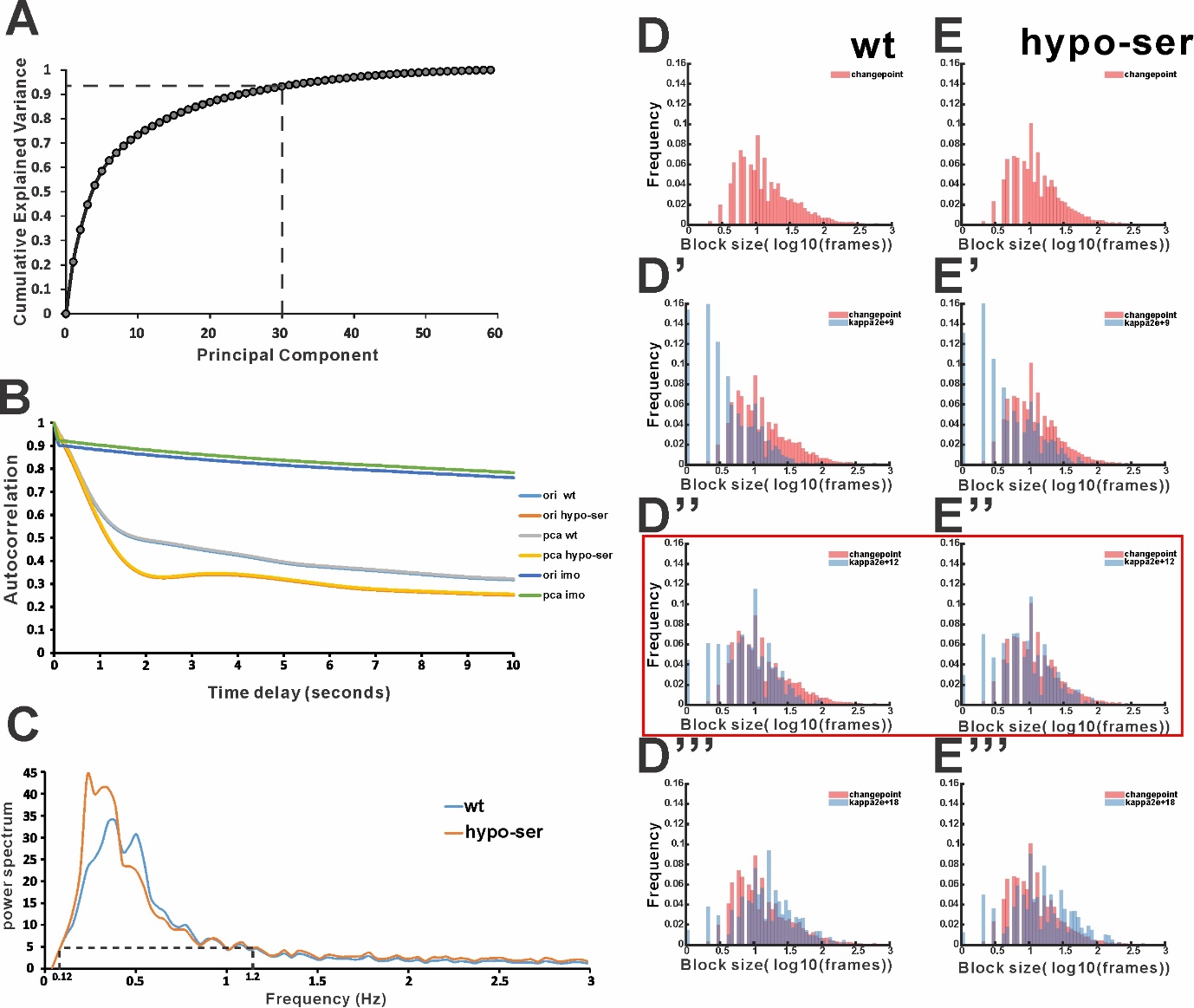
**

**Fig. S3.** A) Cumulative explained variance by the number of principal components. B) Mean autocorrelation of measurements overtime in original posture/muscle activity data (ori) or the first 30 principal component data (pca). imo: immobilized. C) Welch’s power spectral density analysis of frequency components in wild type and hypo-ser datasets. D-E’’’) Block size distributions of changepoint analysis or AR-HMM modeling with various kappa values. Red box highlight the kappa value that was used for further analysis.


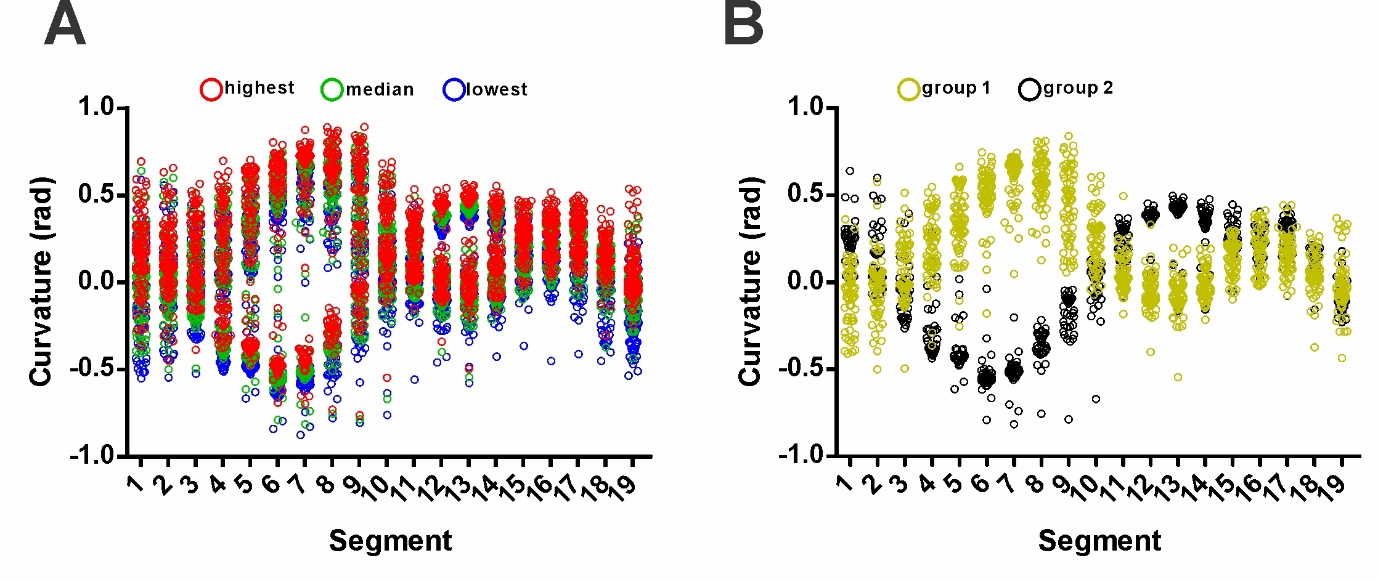
**Fig. S4.** A) Distribution of the posture dynamics of module 10 as in Figure 5A. Each circle represent one sample block. B) Distribution of the median curvature of each segment in module 10 as in A), grouped by their curvature at segments 4-9 and 12-14.


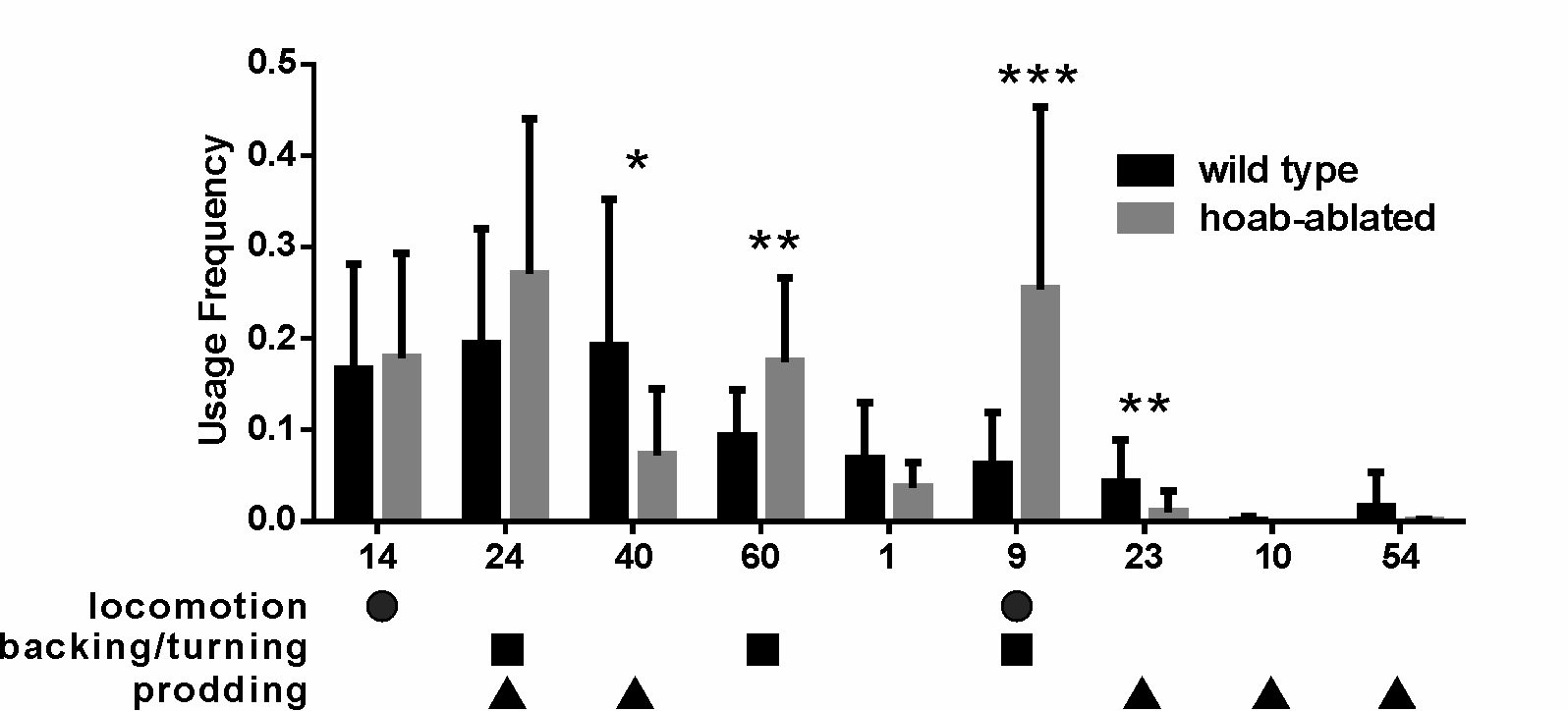


**Fig. S5.** Usage frequencies of major behavioral by wild type and HOA+HOB ablated males. The different shape labels the mating steps that are associated with those modules. The error bars represent standard deviation. p-value is calculated using Mann-Whitney test. ** p<0.01, *** p<0.001. N equals 62 and 11 for wild type and HOA+HOB ablated males, respectively.

**SI References**

1. S. Brenner, The genetics of Caenorhabditis elegans. *Genetics* **77**, 71-94 (1974).

2. H. Schnabel, R. Schnabel, An Organ-Specific Differentiation Gene, pha-1, from Caenorhabditis elegans. *Science* **250**, 686-688 (1990).

3. S. L. Edwards *et al.*, A novel molecular solution for ultraviolet light detection in Caenorhabditis elegans. *PLoS biology* **6**, e198 (2008).

4. J. Hodgkin, H. R. Horvitz, S. Brenner, Nondisjunction Mutants of the Nematode CAENORHABDITIS ELEGANS. *Genetics* **91**, 67-94 (1979).

5. T. R. Gruninger, D. G. Gualberto, L. R. Garcia, Sensory perception of food and insulin-like signals influence seizure susceptibility. *PLoS Genet* **4**, e1000117 (2008).

6. B. LeBoeuf, P. Correa, C. Jee, L. R. García, Caenorhabditis elegans male sensory-motor neurons and dopaminergic support cells couple ejaculation and post-ejaculatory behaviors. *Elife* **3** (2014).

7. P. Anderson, Mutagenesis. *Methods Cell Biol* **48**, 31-58 (1995).

8. D. J. Reiner *et al.*, Behavioral genetics of caenorhabditis elegans unc-103-encoded erg-like K(+) channel. *J Neurogenet* **20**, 41-66 (2006).

9. P. Correa, B. LeBoeuf, L. R. García, C. elegans dopaminergic D2-like receptors delimit recurrent cholinergic-mediated motor programs during a goal-oriented behavior. *PLoS Genet* **8**, e1003015 (2012).

10. M. Granato, H. Schnabel, R. Schnabel, pha-1, a selectable marker for gene transfer in C. elegans. *Nucleic Acids Res* **22**, 1762-1763 (1994).

11. T. R. Gruninger (2008) The Neuromuscular Mechanisms That Coordinate Food Availability With C. elegans Male Sexual Behavior. in *Texas A&M University* (Texas A&M University).

12. T. R. Gruninger, D. G. Gualberto, B. LeBoeuf, L. R. Garcia, Integration of male mating and feeding behaviors in Caenorhabditis elegans. *J Neurosci* **26**, 169-179 (2006).

13. T. Stiernagle (Maintenance of C. elegans. in *WormBook*, ed T. C. e. R. Community (WormBook).

14. A. Vedaldi, K. Lenc, MatConvNet Convolutional Neural Networks for MATLAB. *Mm'15: Proceedings of the 2015 Acm Multimedia Conference* 10.1145/2733373.2807412, 689-692 (2015).

15. A. B. Wiltschko *et al.*, Mapping Sub-Second Structure in Mouse Behavior. *Neuron* **88**, 1121-1135 (2015).

16. M. Basseville, I. V. Nikiforov, *Detection of abrupt changes : theory and application*, Prentice Hall information and system sciences series (Prentice Hall, Englewood Cliffs, N.J., 1993), pp. xxv, 528 p.

17. E. B. Fox, E. B. Sudderth, M. I. Jordan, A. S. Willsky (2008) An HDP-HMM for systems with state persistence. in *Proceedings of the 25th international conference on Machine learning* (Association for Computing Machinery, Helsinki, Finland), pp 312–319.

18. E. Fox, M. Jordan, E. Sudderth, A. Willsky, Sharing features among dynamical systems with beta processes. *Advances in neural information processing systems* **22** (2009).
